# MUC1-C regulates NEAT1 lncRNA expression and paraspeckle formation in cancer progression

**DOI:** 10.1038/s41388-024-03068-3

**Published:** 2024-05-27

**Authors:** Atrayee Bhattacharya, Keyi Wang, Johany Penailillo, Chi Ngai Chan, Atsushi Fushimi, Nami Yamashita, Tatsuaki Daimon, Naoki Haratake, Hiroki Ozawa, Ayako Nakashoji, Keisuke Shigeta, Yoshihiro Morimoto, Masaaki Miyo, Donald W. Kufe

**Affiliations:** 1 Dana-Farber Cancer Institute, Harvard Medical School, Boston, MA USA; 2 Tissue Technologies Unit, Center for Virology and Vaccine Research, Beth Israel Deaconess Medical Center, Harvard Medical School, Boston, MA USA; 3 Breast Surgical Oncology, Breast Oncology Center, The Cancer Institute Hospital of the JFCR, Tokyo, Japan; 4 Department of Gastroenterological Surgery, Graduate School of Medicine, Osaka University, Suita, Osaka 565-0871 Japan

**Keywords:** Cancer stem cells, Non-coding RNAs

## Abstract

The *MUC1* gene evolved in mammals for adaptation of barrier tissues in response to infections and damage. Paraspeckles are nuclear bodies formed on the NEAT1 lncRNA in response to loss of homeostasis. There is no known intersection of MUC1 with NEAT1 or paraspeckles. Here, we demonstrate that the MUC1-C subunit plays an essential role in regulating NEAT1 expression. MUC1-C activates the *NEAT1* gene with induction of the NEAT1_1 and NEAT1_2 isoforms by NF-κB- and MYC-mediated mechanisms. MUC1-C/MYC signaling also induces expression of the SFPQ, NONO and FUS RNA binding proteins (RBPs) that associate with NEAT1_2 and are necessary for paraspeckle formation. MUC1-C integrates activation of *NEAT1* and RBP-encoding genes by recruiting the PBAF chromatin remodeling complex and increasing chromatin accessibility of their respective regulatory regions. We further demonstrate that MUC1-C and NEAT1 form an auto-inductive pathway that drives common sets of genes conferring responses to inflammation and loss of homeostasis. Of functional significance, we find that the MUC1-C/NEAT1 pathway is of importance for the cancer stem cell (CSC) state and anti-cancer drug resistance. These findings identify a previously unrecognized role for MUC1-C in the regulation of NEAT1, RBPs, and paraspeckles that has been co-opted in promoting cancer progression.

## Introduction

Paraspeckles are nuclear condensates formed by the lncRNA NEAT1 and multiple associated RNA binding proteins (RBPs) [1]. The *NEAT1* locus encodes (i) a 3.7 kb NEAT1_1 transcript which is dispensable for paraspeckle formation, and (ii) a 23 kb NEAT1_2 isoform that functions as an essential scaffold for binding of paraspeckle proteins [2]. NEAT1_1 overlaps with the 5’ end of the NEAT1_2 isoform, which is extended by alternative 3’ end processing [2]. NEAT1_1 is polyadenylated, whereas the 3’ end of NEAT1_2 contains a triple-helix structure that protects it from degradation [3, 4]. The proportion of NEAT1_2 to NEAT1_1 dictates the properties of paraspeckles [4], emphasizing the importance of mechanisms regulating expression of the two isoforms [5]. Binding of transcription factors, such as p53, MYC, CEBPB, and others, to the *NEAT1* promoter region activates transcription with isoform switching between NEAT1_1 and NEAT1_2 [6]. Paraspeckle abundance is dependent on NEAT1_2 and not NEAT1_1, which plays a role in cell metabolism [6, 7]. NEAT1_2 includes hydrophobic A, hydrophilic B, and hydrophobic C regions that recruit the splicing factor proline- and glutamine-rich (SFPQ), non-POU domain-containing octamer-binding protein (NONO), fused in sarcoma (FUS), RNA binding motif protein 14 (RBM14), DAZ-associated protein 1 (DAZAP1), HNRNPK and HNRNPH3 RBPs essential for paraspeckle biogenesis [2, 8–10]. Among these, the Drosophila behavior human splicing family members, SFPQ and NONO, form heterodimers involved in pre-mRNA splicing, DNA repair and transcriptional regulation [11]. Oligomerization of SFPQ and NONO also enhances the recruitment of other RBPs, such FUS, that are essential for paraspeckle formation and are of importance in sequestering nuclear RNAs and regulating gene expression [2, 10]. Retention of these RBPs and RNAs contribute to liquid–liquid phase separation (LLPS) of paraspeckles [12].

The *MUC1* gene evolved in mammals to confer adaptation of barrier tissues to infections and damage [13–15]. *MUC1* encodes two subunits that form a heterodimer at the apical membranes of polarized barrier epithelial cells [14, 15]. The extracellular MUC1-N subunit contains glycosylated tandem repeats that extend into the glycocalyx and physically contribute to a protective mucous gel [13–15]. The transmembrane MUC1-C subunit is activated by disruption of homeostasis and drives inflammatory, proliferative, and reprogramming responses that promote wound healing [14, 15]. MUC1 represents a beneficial evolutionary adaptation for survival, which is in principle reversible [15]. However, prolonged activation of MUC1-C in response to chronic inflammation promotes progression to cancer [14, 15]. MUC1-C induces (i) loss of polarity [16], (ii) the epithelial-mesenchymal transition (EMT) [17, 18], and (iii) epigenetic reprogramming by the Polycomb Repressive Complexes (PRC1/2) and COMPASS family of H3K4 methyltransferases [19–23]. MUC1-C also regulates the nucleosome remodeling and deacetylation (NuRD) complex [24] and the SWI/SNF BAF [25] and PBAF [26] chromatin remodeling complexes. In this way, MUC1-C drives global changes in chromatin architecture with increases in accessibility of enhancer-like sequences in stemness-associated genes that contribute to the cancer stem cell (CSC) state [23, 27]. CSCs are dependent on MUC1-C for self-renewal capacity and tumorigenicity [28–33]. Moreover, MUC1-C integrates induction of the CSC state with DNA damage resistance [31, 34, 35] and evasion of anti-tumor immunity [36–38].

MUC1-C regulates gene activation in chromatin by interacting with transcription factors and effectors of epigenetic reprogramming [15]. Paraspeckles regulate gene transcription through RNA-RNA interactions and by sequestering RNAs and proteins. MUC1-C and paraspeckles thus regulate gene expression by distinct mechanisms. There is no known involvement of MUC1-C in integrating regulation of gene expression in chromatin with that in paraspeckles or other subnuclear biomolecular condensates. The present results demonstrate that MUC1-C is necessary for NEAT1 expression in human cancer cells by NF-κB- and MYC-mediated pathways. We show that MUC1-C is also required for induction of SFPQ, NONO, and FUS by MYC-dependent mechanisms. MUC1-C-induced activation of *NEAT1* and *RBP* genes is integrated by recruitment of the BRG1 and PBRM1 components of the PBAF complex and increases in chromatin accessibility. Our findings further demonstrate that MUC1-C is necessary for paraspeckle formation in association with driving cancer progression.

## Results

### MUC1-C is necessary for NEAT1 expression in human cancer cells

**Fig. 1:**
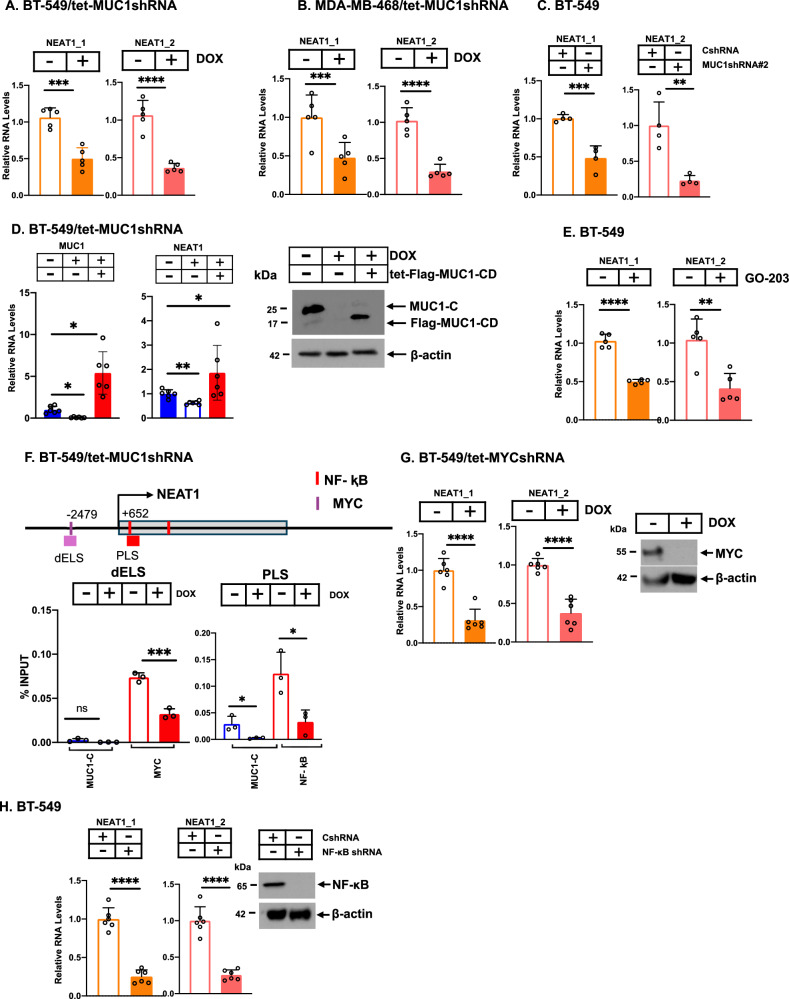
MUC1-C activates NEAT1 expression by MYC- and NF-κB-mediated mechanisms. BT-549/tet-MUC1shRNA (**A**) and MDA-MB-468/tet-MUC1shRNA (**B**) cells treated with vehicle or DOX for 7 days were analyzed for NEAT1_1 and NEAT1_2 transcripts by qRT-PCR using primers listed in Supplementary Table S1. The results (mean±SD of 5 determinations) are expressed as relative levels compared to that obtained for vehicle-treated cells (assigned a value of 1). **C** BT-549/CshRNA and BT-549/MUC1shRNA#2 cells were analyzed for NEAT1_1 and NEAT1_2 transcripts by qRT-PCR. The results (mean±SD of at least 3 independent biological replicates) are expressed as relative levels compared to that obtained for CshRNA-expressing cells (assigned a value of 1). **D** BT-549/tet-MUC1shRNA cells expressing a tet-MUC1-CD vector were treated with vehicle or DOX for 7 days and analyzed for NEAT1_1 and NEAT1_2 transcripts by qRT-PCR. The results (mean±SD of at least 3 independent biological replicates) are expressed as relative levels compared to that obtained for vehicle-treated cells (assigned a value of 1)(left). Lysates were immunoblotted with antibodies against the indicated proteins (right). **E** BT-549 cells treated with vehicle or 5 μM GO-203 for 48 h were analyzed for NEAT1_1 and NEAT1_2 transcripts by qRT-PCR. The results (mean±SD of 5 determinations) are expressed as relative levels compared to that obtained for vehicle-treated cells (assigned a value of 1). **F** Schema of the *NEAT1* gene with highlighting of the dELS and PLS regions. Soluble chromatin from BT-549/tet-MUC1shRNA cells treated with vehicle or DOX for 7 days was precipitated with anti-MUC1-C and anti-MYC (left) or with anti-MUC1-C and anti-NF-κB p65 (right). The DNA samples were amplified by qPCR with primers for the *NEAT1* dELS region (left) and the PLS region (right). The results (mean ± SD of 3 determinations) are expressed as percent input. **G** BT-549/tet-MYCshRNA cells treated with vehicle or DOX for 7 days were analyzed for NEAT1_1 and NEAT1_2 transcripts by qRT-PCR. The results (mean±SD of 4 determinations) are expressed as relative levels compared to that obtained for vehicle-treated cells (assigned a value of 1)(left). Lysates were immunoblotted with antibodies against the indicated proteins (right). **H** BT-549/CshRNA and BT-549/NF-κBshRNA cells were analyzed for NEAT1_1 and NEAT1_2 transcripts by qRT-PCR. The results (mean ± SD of 6 determinations) are expressed as relative levels compared to that obtained for CshRNA-expressing cells (assigned a value of 1)(left). Lysates were immunoblotted with antibodies against the indicated proteins (right).

Few insights are available regarding involvement of MUC1-C in the regulation of lncRNAs in cancer [7, 39, 40]. Here, silencing MUC1-C in BT-549 TNBC cells with a tet-inducible MUC1shRNA (Supplementary Fig. S1A) decreased expression of the ~3.7 kb NEAT1_1 and ~23 kb NEAT1_2 transcripts (Fig. 1A). As a control, DOX treatment of BT-549/tet-CshRNA cells had little if any effect on NEAT1 expression (Supplementary Fig. S1B). Similar results were obtained in MDA-MB-468 TNBC (Fig. 1B; Supplementary Fig. S1C), MDA-MB-436 TNBC (Supplementary Fig. S1D) and DU-145 CRPC (Supplementary Fig. S1E) cells, indicating that MUC1-C-dependent NEAT1 expression is observed across cancer cell lineages. In excluding off-target effects, silencing MUC1-C with a second MUC1shRNA#2 similarly suppressed NEAT1_1 and NEAT1_2 transcripts (Fig. 1C; Supplementary Fig. S1F). To confirm MUC1-C dependence, MUC1-C downregulation was rescued with the MUC1-C cytoplasmic domain (tet-Flag-MUC1-CD), which restored NEAT1 RNA levels (Fig. 1D). The MUC1-C 72 aa cytoplasmic domain contains a CQC motif indispensable for MUC1-C homodimerization and function [14, 41, 42]. Targeting the MUC1-C CQC motif with the GO-203 inhibitor suppressed NEAT1_1 and NEAT1_2 transcripts (Fig. 1E). We also found that silencing MUC1-C decreases the synthesis of nascent NEAT1 transcripts (Supplementary Fig. S1G), indicating that MUC1-C contributes to *NEAT1* transcription. *NEAT1* includes a MYC binding motif at a distal enhancer-like sequence (dELS) region and a NF-κB binding motif in a promoter-like sequence (PLS) residing upstream and downstream to the transcription start site (TSS), respectively (Fig. 1F) [43]. The MUC1-C cytoplasmic domain binds directly to MYC and NF-κB p65 in regulating their target genes [14, 24, 42, 44]. ChIP studies demonstrated occupancy of (i) MYC on the dELS, and (ii) MUC1-C and NF-κB on the PLS (Fig. 1F). Moreover, silencing MUC1-C significantly decreased (i) MYC occupancy on the dELS, and (ii) NF-κB occupancy on the PLS (Fig. 1F). In concert with these results, (i) silencing MYC (Fig. 1G) and targeting NF-κB genetically (Fig. 1H) or pharmacologically with the BAY-11 inhibitor (Supplementary Fig. S1H) decreased NEAT1_1 and NEAT1_2 transcripts, and (ii) targeting MYC and NF-κB in combination more effectively decreased NEAT1 expression than either alone (Supplementary Fig. S1I), indicating that MUC1-C regulates NEAT1 expression by MYC- and NF-κB-mediated mechanisms. These findings indicate that MUC1-C regulates the *NEAT1* gene at pELS and PLS regions, which control transcription of both NEAT1_1 and NEAT1_2.

### MUC1-C and NEAT1 form an auto-regulatory pathway that drives inflammatory and EMT gene signatures

**Fig. 2:**
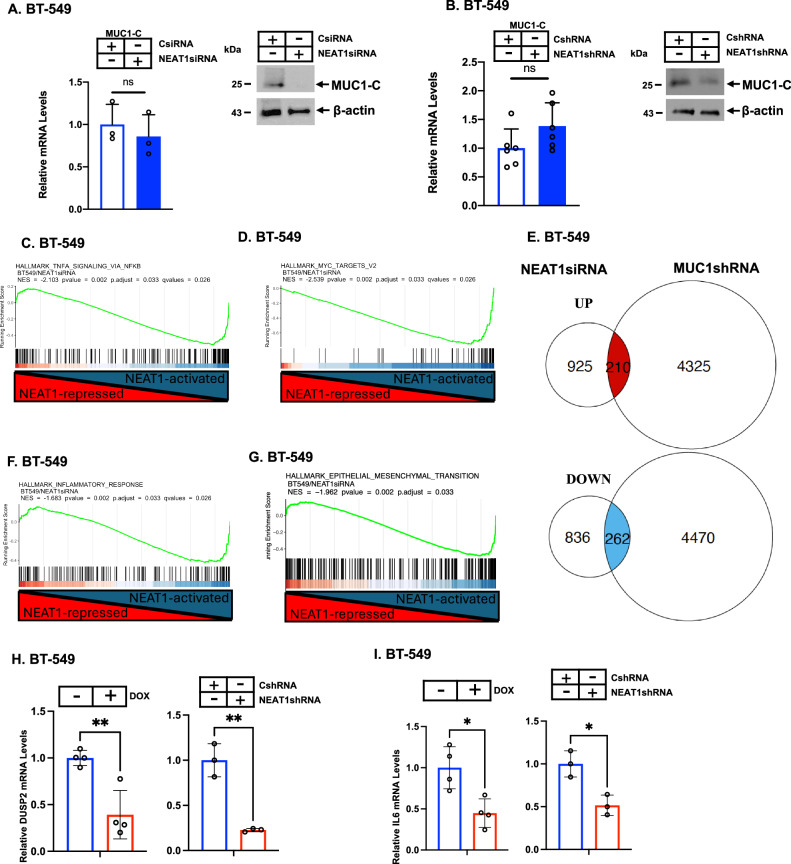
NEAT1 regulates MUC1-C expression in an auto-inductive pathway. **A** BT-549/CsiRNA and BT-549/NEAT1siRNA cells were analyzed for MUC1-C transcripts by qRT-PCR. The results (mean±SD of 3 biological replicates) are expressed as relative levels compared to that obtained for CsiRNA cells (assigned a value of 1)(left). Lysates were immunoblotted with antibodies against the indicated proteins (right). **B** BT-549/CshRNA and BT-549/NEAT1shRNA cells were analyzed for MUC1-C transcripts by qRT-PCR. The results (mean±SD of 3 independent biological replicates) are expressed as relative levels compared to that obtained for CshRNA cells (assigned a value of 1)(left). Lysates were immunoblotted with antibodies against the indicated proteins (right). **C**, **D** GSEA of RNA-seq data from NEAT1siRNA vs CsiRNA cells using the indicated HALLMARK gene signatures. **E** Venn diagrams of common downregulated and upregulated genes in NEAT1- and MUC1-C-silenced cells. **F**, **G** GSEA of RNA-seq data from NEAT1siRNA vs CsiRNA cells using the indicated HALLMARK gene signatures. **H**, **I** BT-549/CshRNA, BT-549/tet-MUC1shRNA cells treated with vehicle or DOX for 7 days, and BT-549/NEAT1shRNA cells were analyzed for DUSP2 (**H**) and IL-6 (**I**) transcripts by qRT-PCR. The results (mean ± SD of 3 independent biological replicates) are expressed as relative levels compared to that obtained for DOX- and CshRNA cells (assigned a value of 1).

Downregulation of NEAT1_1 and NEAT1_2 with a NEAT1siRNA or a different NEAT1shRNA (Supplementary Fig. S2A and S2B) demonstrated that NEAT1 is dispensable for the regulation of MUC1-C mRNA levels but is necessary for expression of the MUC1-C protein (Fig. 2A, B). These results were extended by demonstrating that silencing NEAT1 decreases stability of the MUC1-C protein (Supplementary Fig. S2C), in support of a MUC1-C/NEAT1 auto-inductive pathway in which (i) MUC1-C drives *NEAT1* transcription and (ii) NEAT1 regulates MUC1-C by a post-transcriptional mechanism. To assess regulation of transcriptomes by NEAT1, we performed RNA-seq on BT-549 cells with NEAT1 silencing and identified 1098 downregulated and 1135 upregulated genes (Supplementary Fig. S2D). GSEA demonstrated that NEAT1 associates with regulation of the HALLMARK TNFA SIGNALING VIA NFKB and HALLMARK MYC TARGETS V2 gene signatures (Fig. 2C and D). Using these signatures, comparison of RNA-seq data from NEAT1- and MUC1-C-silenced cells identified 210 and 262 common downregulated and upregulated genes, respectively (Fig. 2E). GSEA also demonstrated that MUC1-C and NEAT1 regulate common sets of genes in the HALLMARK TNFA SIGNALING VIA NFKB (Supplementary Fig. S2F), HALLMARK MYC TARGETS V2 (Supplementary Fig. S2G), HALLMARK INFLAMMATORY RESPONSE (Fig. 2F; Supplementary Fig. S2H) and the HALLMARK MESENCHYMAL TRANSITION (Fig. 2G; Supplementary Fig. S2I) gene signatures. Among these genes, we confirmed as selected examples that MUC1-C and NEAT1 regulate expression of the dual phosphatase DUSP2, which is a target of NF-κB and MYC (Fig. 2H), and (ii) IL-6, which is linked to chronic inflammation and EMT (Fig. 2I). These findings indicated that MUC1-C and NEAT1 form an auto-inductive pathway that (i) regulates NF-κB and MYC target genes and (ii) associates with chronic inflammation and EMT.

### MUC1-C regulates expression of NEAT1 RBPs

**Fig. 3:**
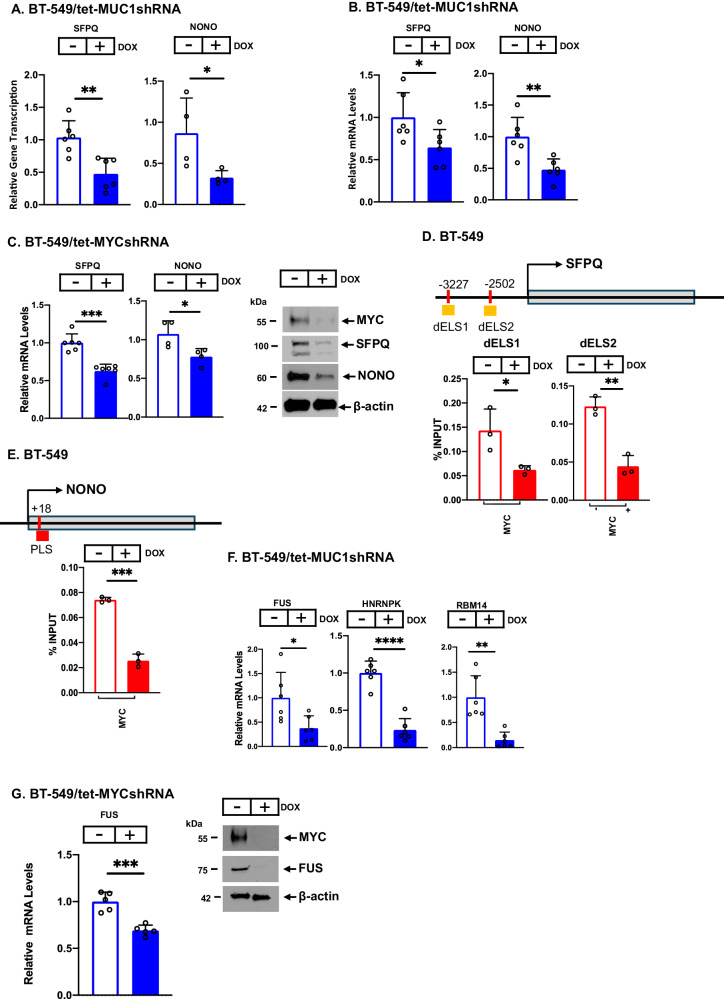
MUC1-C regulates SFPQ, NONO, and FUS expression by MYC-dependent mechanisms. **A** BT-549/tet-MUC1shRNA cells treated with vehicle or DOX for 7 days were analyzed for nascent *SFPQ* and *NONO* transcription. The results (mean±SD of at least 3 independent biological replicates) are expressed as relative gene transcription compared to that obtained in vehicle-treated cells (assigned a value of 1). **B** BT-549/tet-MUC1shRNA cells treated with vehicle or DOX for 7 days were analyzed for the indicated transcripts by qRT-PCR. The results (mean±SD of 3 biological replicates) are expressed as relative mRNA levels compared to that obtained for vehicle-treated cells (assigned a value of 1). **C** BT-549/tet-MYCshRNA cells treated with vehicle or DOX for 7 days were analyzed for SFPQ and NONO transcripts by qRT-PCR (left). The results (mean±SD of at least 3 replicates) are expressed as relative levels compared to that obtained for vehicle-treated cells (assigned a value of 1). Lysates were immunoblotted with antibodies against the indicated proteins (right). **D** Schema of the *SFPQ* gene with highlighting of the dELS1 and dELS2 regions. Soluble chromatin from BT-549/tet-MUC1shRNA cells treated with vehicle or DOX for 7 days was precipitated with anti-MYC. The DNA samples were amplified by qPCR with primers for the *SFPQ* dELS1 and dELS2 regions. The results (mean ± SD of 3 replicates) are expressed as percent input. **E** Schema of the *NONO* gene with highlighting of the PLS region. Soluble chromatin from BT-549/tet-MUC1shRNA cells treated with vehicle of DOX for 7 days was precipitated with anti-MYC. The DNA samples were amplified by qPCR with primers for the *NONO* PLS region. The results (mean ± SD of 3 replicates) are expressed as percent input. **F** BT-549/tet-MUC1shRNA cells treated with vehicle or DOX for 7 days were analyzed for the indicated transcripts by qRT-PCR. The results (mean±SD of 3 biological replicates) are expressed as relative mRNA levels compared to that obtained for vehicle-treated cells (assigned a value of 1). **G** BT-549/tet-MYCshRNA cells treated with vehicle or DOX for 7 days were analyzed for FUS transcripts by qRT-PCR. The results (mean±SD of at least 3 biological replicates) are expressed as relative mRNA levels compared to that obtained for vehicle-treated cells (assigned a value of 1) (left). Lysates were immunoblotted with antibodies against the indicated proteins (right).

Among common downregulated genes in MUC1-C- and NEAT1-silenced cells, we identified *SFPQ*, which encodes a NEAT1_2 RBP with intrinsically disordered regions that contribute to biomolecular condensates [45]. Oligomerization of SFPQ with NONO on NEAT1_2 is required for forming mature paraspeckles [2, 9, 10]. Silencing MUC1-C downregulated *SFPQ* and *NONO* gene transcription (Fig. 3A) and mRNA levels (Fig. 3B; Supplementary Figs. S3A and S3B). In addition, silencing MYC decreased SFPQ and NONO expression (Fig. 3C), whereas downregulation of NF-κB had no significant effect (Supplementary Fig. S3C). Along these lines, MYC binding motifs were identified in *SFPQ* distal enhancer-like signature 1 (dELS1) and dELS2 regions (Fig. 3D). MYC occupancy of those regions was found to be MUC1-C-dependent (Fig. 3D). A MYC binding motif was also identified in a *NONO* PLS region of which occupancy by MYC was decreased by MUC1-C silencing (Fig. 3E). Oligomerization of SFPQ and NONO on NEAT1_2 recruits additional RBPs, such as FUS, RBM14 and HNRNPK, that further contribute to paraspeckle formation [2, 9, 10]. We found that MUC1-C is necessary for expression of FUS, RBM14 and HNRNPK (Fig. 3F; Supplementary Figs. S3D and S3E). Moreover, MYC was necessary for FUS (Fig. 3G), but not HNRNPK and RBM14, expression (Supplementary Fig. S3F). Together, these findings supported involvement of (i) the MUC1-C→MYC pathway in inducing SFPQ, NONO and FUS, and (ii) MUC1-C in driving RBM14 and HNRNPK by a MYC-independent mechanism.

### MUC1-C regulates expression of NEAT1 and RBP-encoding genes by PBAF-mediated chromatin remodeling

**Fig. 4:**
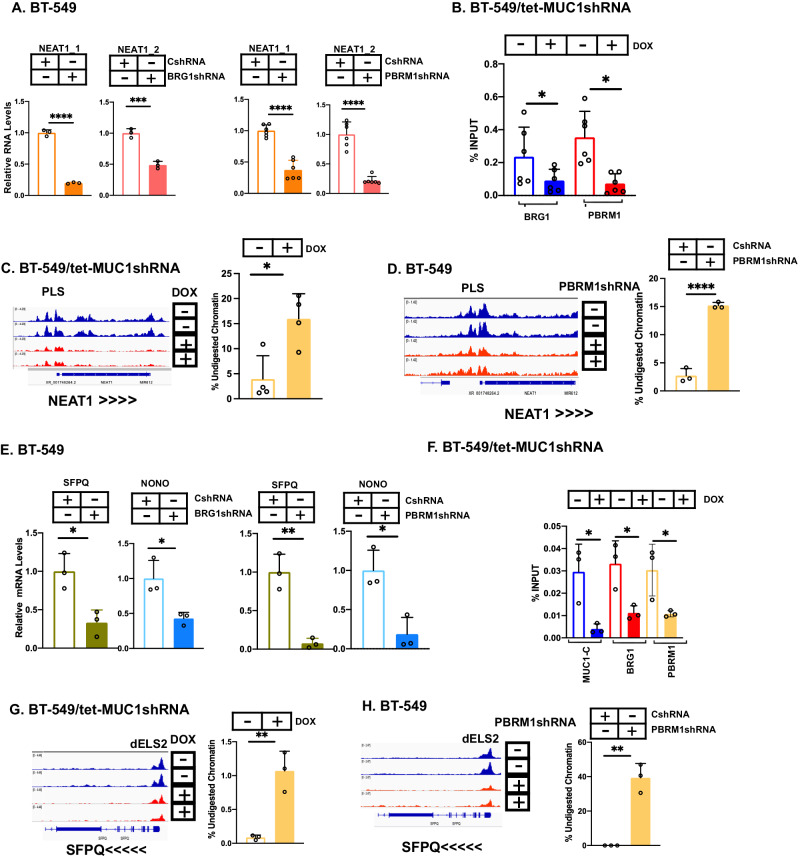
MUC1-C activates *NEAT1* and *RBP* genes by PBAF-mediated increases in chromatin accessibility. **A** BT-549 cells expressing a CshRNA, BRG1shRNA or PBRM1shRNA were analyzed for NEAT1_1 and NEAT1_2 transcripts by qRT-PCR. The results (mean±SD of three independent biological replicates) are expressed as relative RNA levels compared to that obtained for CshRNA-expressing cells (assigned a value of 1). **B** Soluble chromatin from BT-549/tet-MUC1shRNA cells treated with vehicle or DOX for 7 days was precipitated with anti-BRG1 and anti-PBRM1. The DNA samples were amplified by qPCR with primers for the *NEAT1* PLS region. The results (mean ± SD of 3 biological replicates) are expressed as percent input. **C** Genome browser snapshot of ATAC-seq data from the *NEAT1* PLS in BT-549/tet-MUC1shRNA cells treated with vehicle or DOX for 7 days (left). Chromatin was analyzed for accessibility by nuclease digestion (right). The results (mean ± SD of at least 3 biological replicates) are expressed as % undigested chromatin. **D** Genome browser snapshot of ATAC-seq data from the *NEAT1* PLS in BT-549/CshRNA and BT-549/PBRM1shRNA cells (left). Chromatin was analyzed for accessibility by nuclease digestion (right). The results (mean ± SD of 3 replicates) are expressed as % undigested chromatin. **E** BT-549 cells expressing a CshRNA, BRG1shRNA or PBRM1shRNA were analyzed for SFPQ and NONO transcripts by qRT-PCR. The results (mean±SD of 3 independent replicates) are expressed as relative RNA levels compared to that obtained for CshRNA-expressing cells (assigned a value of 1). **F** Soluble chromatin from BT-549/tet-MUC1shRNA cells treated with vehicle or DOX for 7 days was precipitated with anti-MUC1-C, anti-BRG1, and anti-PBRM1. The DNA samples were amplified by qPCR with primers for the *SFPQ* dELS2 region. The results (mean ± SD of three independent biological replicates) are expressed as percent input. **G**. Genome browser snapshot of ATAC-seq data from the *SFPQ* dELS2 in BT-549/tet-MUC1shRNA cells treated with vehicle or DOX for 7 days (left). Chromatin was analyzed for accessibility by nuclease digestion (right). The results (mean ± SD of 3 replicates) are expressed as % undigested chromatin. **H** Genome browser snapshot of ATAC-seq data from the *SFPQ* dELS2 in BT-549/CshRNA and BT-549/PBRM1shRNA cells (left). Chromatin was analyzed for accessibility by nuclease digestion (right). The results (mean ± SD of 3 replicates) are expressed as % undigested chromatin.

MUC1-C activates the SWI/SNF BAF and PBAF chromatin remodeling complexes [15, 25, 26, 42]. Here, silencing BRG1, which is shared with BAF and PBAF, suppressed NEAT1_1 and NEAT1_2 transcripts (Fig. 4A). Silencing the PBRM1 component of PBAF, but not the ARID1A subunit of BAF, also decreased NEAT1_1 and NEAT1_2 expression (Fig. 4A; Supplementary Fig. S4A), indicating that PBAF plays a role in activating the *NEAT1* gene. Analysis of the *NEAT1* PLS region demonstrated that silencing MUC1-C is associated with decreases in (i) BRG1 and PBRM1 occupancy (Fig. 4B), and (ii) chromatin accessibility (Fig. 4C). Silencing PBRM1 also decreased chromatin accessibility of the *NEAT1* PLS (Fig. 4D), in further support for involvement of the PBAF complex. As identified for NEAT1, we found that BRG1 and PBRM1 regulate SFPQ and NONO expression (Fig. 4E). Silencing MUC1-C decreased BRG1 and PBRM1 occupancy (Fig. 4F) and chromatin accessibility (Fig. 4G) of the *SFPQ* dELS2. Similar effects of MUC1-C silencing were observed for the *NONO* PLS region (Supplementary Figs. S4B and S4C). In addition, silencing PBRM1 downregulated chromatin accessibility of the *SFPQ* dELS2 (Fig. 4H) and *NONO* PLS (Supplementary Fig. S4D) regions. Silencing MUC1-C and PBRM1 was also associated with decreases in chromatin accessibility of the *FUS* PLS (Supplementary Figs. S4E and S4F). Consistently, BRG1 and PBRM1 were necessary for FUS expression (Supplementary Fig. S4G). These findings indicated that MUC1-C-mediated induction of PBAF integrates activation of *NEAT1* with genes encoding the SFPQ, NONO, and FUS RBPs.

### MUC1-C forms nuclear complexes with NEAT1_2 RBPs

**Fig. 5:**
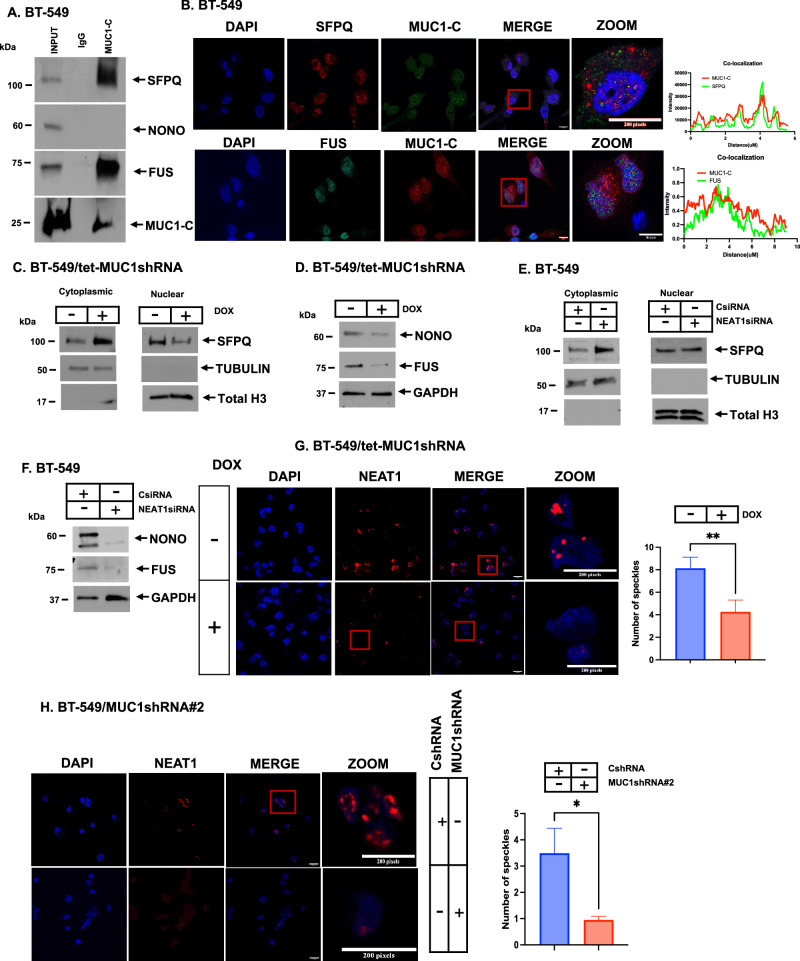
MUC1-C-induced regulation of RBP proteins. **A** Lysates of BT-549 cells were immunoprecipitated with a control IgG or anti-MUC1-C. The input lysate and precipitates were immunoblotted with antibodies against the indicated proteins. **B** Immunofluorescence staining of MUC1-C, SFPQ, and FUS in BT-549 cells. Nuclei were stained with DAPI. The enlarged inset images and colocalization analyses are on the right. Pearson’s coefficients of colocalization: MUC1-C + SFPQ = 0.625, MUC1-C + FUS = 0.594. **C** Cytoplasmic and nuclear lysates from BT-549/CsiRNA and BT-549/NEAT1siRNA cells were immunoblotted with antibodies against the indicated proteins. **D** Total cell lysates of BT-549/tet-MUC1shRNA cells treated with vehicle or DOX for 7 days were immunoblotted with antibodies against the indicated proteins. **E** Cytoplasmic and nuclear lysates from BT-549/CsiRNA and BT-549/NEAT1siRNA were immunoblotted with antibodies against the indicated proteins. **F** Total cell lysates from BT-549/CsiRNA and BT-549/NEAT1siRNA cells were immunoblotted with antibodies against the indicated proteins. **G** Representative NEAT1 RNA-FISH images of BT-549/tet-MUC1shRNA cells treated with vehicle or DOX for 7 days with highlighting of red NEAT1 foci. **H** Representative NEAT1 RNA-FISH images of BT-549/CshRNA and BT-549/MUC1shRNA#2 cells with highlighting of red NEAT1 foci.

Assessment of MUC1-C expression by IHC of TNBC core biopsies demonstrated staining in the cell membrane and cytoplasm [38, 46]. MUC1-C is also expressed in the nucleus and localizes to chromatin [33, 47]. Here, analysis of anti-MUC1-C nuclear precipitates from TNBC cells by mass spectroscopy identified the (i) NEAT1_2 FUS and RBM14 RBPs, and (ii) components of the BAF chromatin remodeling complex, SMARCC1, SMARCC2, ARID1A, SMARC4A/BRG1 and ARID1B that are integral to paraspeckles [9, 25] (Supplementary Fig. S5A). We also found that MUC1-C associates with the (i) nucleoporin 62 (NUP62), NUP358/RANBP2, NUP214, NUP93, NUP88, NUP205 and NUP98 components of the nuclear pore complex (NPC), and (ii) WTAP, VIRMA and ZC3H13 effectors of RNA m6A methylation [48, 49] (Supplementary Fig. S5A). In focusing on NEAT1_2 RBPs, nuclear coimmunoprecipitation studies in BT-549 cells demonstrated that MUC1-C associates with SFPQ and FUS, but not NONO (Fig. 5A). Immunofluorescence microscopy further showed that nuclear MUC1-C colocalizes with SFPQ and FUS (Fig. 5B). In addition, coimmunoprecipitation studies and immunofluorescence microscopy of MDA-MB-468 cells identified MUC1-C complexes with SFPQ and FUS, as well as NONO (Supplementary Fig. S5B and S5C). We also found that MUC1-C localizes with PSPC1, another marker of paraspeckles (Supplementary Fig. S5D). NEAT1_2 is necessary for the retention of SFPQ, NONO, and FUS in paraspeckles [10]. Downregulation of MUC1-C was associated with increases in cytoplasmic SFPQ and decreases in nuclear SFPQ levels (Fig. 5C). By contrast, silencing MUC1-C downregulated total levels of NONO and FUS expression (Fig. 5D). Silencing NEAT1 had similar effects; that is increases in SFPQ in the cytoplasm (Fig. 5E) and decreases in total levels of NONO and FUS (Fig. 5F), indicating that downregulation of MUC1-C and thereby NEAT1_2 regulates SFPQ, FUS and NONO expression. In concert with these results, inducible MUC1-C silencing decreased nuclear NEAT1 paraspeckles (Fig. 5G; Supplementary Fig. S5F). This dependence on MUC1-C for forming paraspeckles was confirmed with stable silencing using MUC1shRNA#2 (Fig. 5H). These findings indicated that (i) MUC1-C forms complexes with NEAT1_2, SFPQ and FUS in paraspeckles, and (ii) targeting MUC1-C and NEAT1_2 disrupts paraspeckle formation in association with localization of SFPQ in the cytoplasm and loss of NONO and FUS expression.

### MUC1-C/NEAT1 pathway confers chemoresistance

**Fig. 6:**
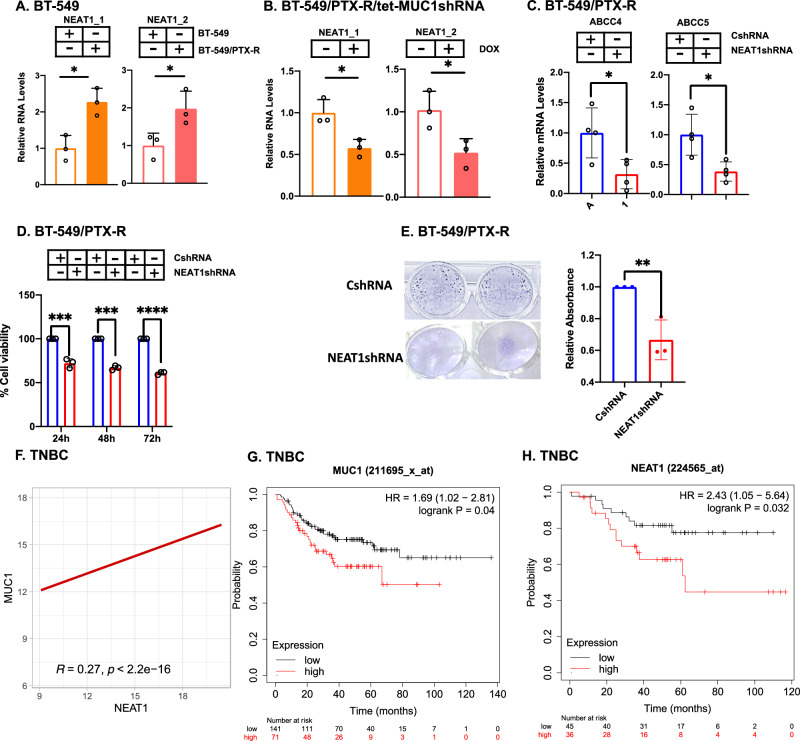
MUC1-C/NEAT1 pathway contributes to chemoresistance. **A** BT-549 and BT-549/PTX-R cells were analyzed for NEAT1_1 and NEAT1_2 transcripts by qRT-PCR. The results (mean±SD of three replicates) are expressed as relative levels compared to that obtained for BT-549 cells (assigned a value of 1). **B** BT-549/PTX-R cells expressing tet-MUC1shRNA were treated with vehicle or DOX for 7 days and analyzed for NEAT1_1 and NEAT1_2 transcripts by qRT-PCR. The results (mean±SD of three replicates) are expressed as relative levels compared to that obtained for vehicle-treated cells (assigned a value of 1). **C** BT-549/PTX-R cells expressing CshRNA or NEAT1shRNA were analyzed for ABCC4 and ABCC5 transcripts by qRT-PCR. The results (mean±SD of three independent replicates) are expressed as relative levels compared to that obtained for CshRNA cells (assigned a value of 1). **D** BT-549/PTX-R cells expressing CshRNA or NEAT1shRNA were treated with 1 nM PTX for 24, 48, and 72 h. Cell viability was assessed by Alamar blue assay. The results (mean ± SD of 3 biologic replicates each with 5 determinations) are expressed as relative levels compared to that obtained for CshRNA cells (assigned a value of 1). **E** BT-549/PTX-R cells expressing CshRNA or NEAT1shRNA treated with PTX were analyzed for colony formation. Shown are representative photomicrographs of stained colonies (left). The results (mean±SD of three biologic replicates) are expressed as relative absorbance compared to that for untreated cells (assigned a value of 1) (right). **F** Analysis of the TCGA BRCA cohort assessing the correlation of MUC1 with NEAT1 in patient samples. Disease-free survival of patients with grade 3 TNBC tumors treated with cytotoxic chemotherapy expressing high vs low levels of MUC1 (**G**) and NEAT1 (**H**).

MUC1-C promotes resistance of cancer cells to cytotoxic and targeted agents [14, 15, 42]; whereas less is known regarding involvement of NEAT1 in the drug-resistant phenotype. In this regard, studies of BT-549 cells selected for resistance to paclitaxel (PTX) [18] demonstrated that BT-549/PTX-R cells have increased NEAT1_1 and NEAT1_2 levels compared to parental BT-549 cells (Fig. 6A). Moreover, targeting MUC1-C in BT-549/PTX-R cells downregulated NEAT1_1 and NEAT1_2 expression (Fig. 6B; Supplementary Fig. S6A). Of functional significance, silencing NEAT1 in BT-549 and BT-549/PTX-R cells decreased expression of the ABC transporters ABCC4/5 that promote PTX efflux (Fig. 6C; Supplementary Fig. S6B) and increased PTX sensitivity (Fig. 6D; Supplementary Fig. S6C). By extension, similar results were obtained in DU-145 cells resistant to docetaxel (DTX); that is, (i) NEAT1_1 and NEAT1_2 levels were increased in DU-145/DTX-R cells compared to parental DU-145 cells (Supplementary Fig. S6D), (ii) targeting MUC1-C in DU-145/DTX-R cells decreased NEAT1_1 and NEAT1_2 expression (Supplementary Fig. S6E), and (iii) silencing NEAT1 downregulated expression of the ABCC4/5 transporters (Supplementary Fig. S6F). We also observed increased DTX sensitivity with silencing of NEAT1 in DU-145/DTX-R cells (Supplementary Fig. S6G). As further support for NEAT1 dependency, NEAT1 knockdown in BT-549/PTX-R and DU-145/DTX-R cells decreased their capacity for clonogenic survival (Fig. 6E; Supplementary Fig. S6H). In assessing whether these results extend to TNBC tumors, we found that MUC1 significantly associates with NEAT1 expression (Fig. 6F). Moreover, analysis of patients with grade 3 TNBCs treated with cytotoxic anti-cancer agents demonstrated that those with MUC1-high (Fig. 6G) and NEAT1-high (Fig. 6H) tumors had significantly decreased relapse-free survival, supporting involvement of the MUC1-C/NEAT1 pathway in conferring chemoresistance.

### MUC1-C/NEAT1 pathway promotes the CSC state

**Fig. 7:**
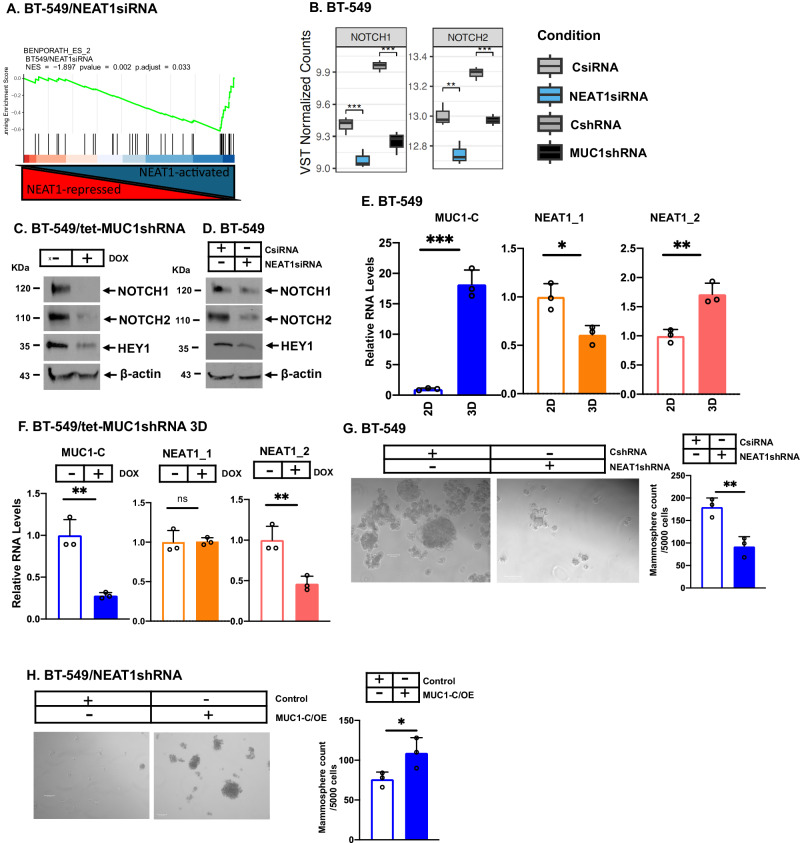
MUC1-C/NEAT1 pathway drives the CSC state. **A** RNA-seq was performed in triplicate on BT-549/NEAT1siRNA and BT-549/CsiRNA cells. GSEA was performed using the BENPORATH ES 2 gene signature. **B** Candidate box plots showing common NEAT1 and MUC1-C driven stemness genes from RNA-seq data. **C** Lysates of BT-549/tet-MUC1shRNA cells treated with vehicle or DOX for 7 days were immunoblotted with antibodies against the indicated proteins. **D** Lysates from BT-549/CsiRNA and BT-549/NEAT1siRNA cells were immunoblotted with antibodies against the indicated proteins. **E** BT-549 cells grown as monolayers in 2D culture and as tumorspheres in 3D culture were analyzed for the indicated RNA levels by qRT-PCR. The results (mean±SD of three replicates) are expressed as relative levels compared to that obtained for BT-549 2D cells (assigned a value of 1). **F** BT-549/tet-MUC1shRNA 3D cells treated with vehicle or DOX for 7 days were analyzed for the indicated RNA levels. The results (mean±SD of three replicates) are expressed as relative levels compared to that obtained for vehicle-treated cells (assigned a value of 1). **G** BT-549/CshRNA 3D and BT-549/NEAT1shRNA 3D cells were analyzed for tumorsphere formation. Photomicrographs are shown for the tumorspheres (bar represents 100 μm; left). The results (mean ± SD of three determinations) are expressed as tumorsphere number (right). **H** BT-549/NEAT1shRNA 3D cells transfected with an empty or MUC1-C-expressing vector were analyzed for tumorsphere formation. Photomicrographs are shown for the tumor spheres (bar represents 100 μm; left). The results (mean ± SD of three determinations) are expressed as tumorsphere number (right).

MUC1-C integrates the CSC state and treatment resistance [14, 15, 42]. Less is known about dependency of CSCs on NEAT1 [50–52]. As reported for MUC1-C [14, 15, 42], silencing NEAT1 was associated with suppressing the BENPORATH ES gene signature (Fig. 7A), derived from embryonic stem cells and advanced carcinomas [53]. We found that MUC1-C and NEAT1 regulate common sets of BENPORATH ES genes, which included those encoding the NOTCH1 and NOTCH2 stemness factors (Fig. 7B; Supplementary Figs. S7A and S7B). Downregulating MUC1-C (Fig. 7C) and NEAT1 (Fig. 7D) also decreased expression of the NOTCH1 and NOTCH2 proteins and their downstream target HEY1. NEAT1_2, but not NEAT1_1, is essential for paraspeckle formation [2]. Silencing MUC1-C and NEAT1 decreased cell surface expression of the CD44 stemness marker as determined by flow cytometry (Supplementary Fig. S7D, E). Analysis of BT-549 cells grown as monolayers in 2D culture and as tumorspheres in 3D culture further demonstrated upregulation of MUC1-C in association with decreases in NEAT1_1 and increases in NEAT1_2 transcripts (Fig. 7E). In addition, silencing MUC1-C in BT-549 3D cells downregulated NEAT1_2, but not NEAT1_1, transcripts (Fig. 7F). In support of the MUC1-C/NEAT1 auto-regulatory pathway in 3D cells, (i) silencing NEAT1 suppressed MUC1-C expression (Supplementary Fig. S7C) and (ii) silencing MUC1-C and NEAT1 downregulated cell surface CD44 expression (Supplementary Fig. S7D and S7E). As reported for MUC1-C [26, 28, 37, 38], silencing NEAT1 suppressed clonogenic survival (Supplementary Fig. S7F) and tumorsphere formation (Fig. 7G; Supplementary Fig. S7G). Additionally, overexpression of MUC1-C in NEAT1-silenced cells (Supplementary Fig. S7H) rescued the loss of capacity for clonogenic survival (Supplementary Fig. S7I) and tumorsphere formation (Fig. 7H), indicating that the NEAT1_2 isoform is of importance for the stemness phenotype and that MUC1-C/NEAT1_2 signaling drives CSC clonogenicity and self-renewal.

## Discussion

**Fig. 8:**
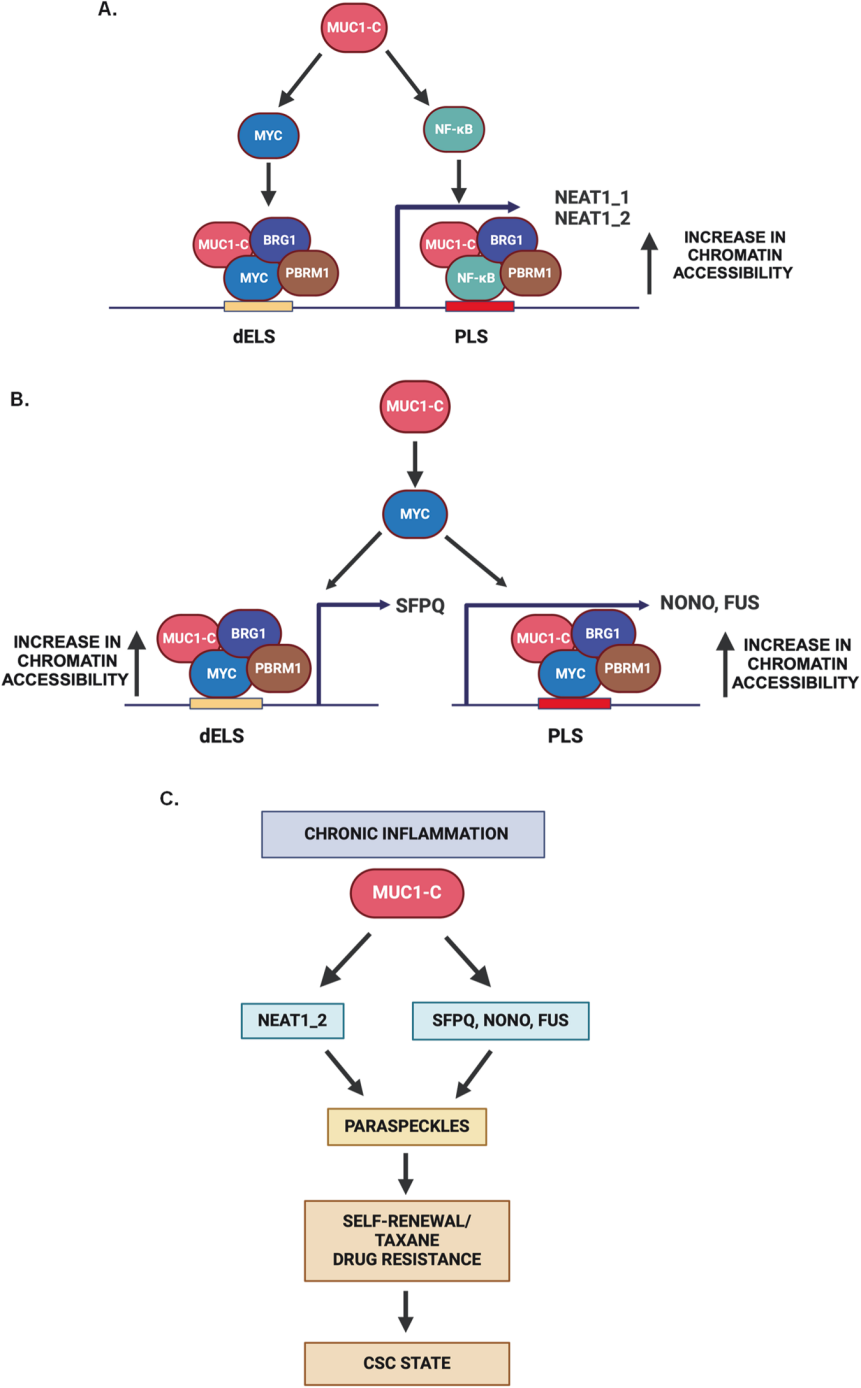
Schema depicting MUC1-C-mediated regulation of *NEAT1* and NEAT1-binding proteins. **A** MUC1-C binds directly to NF-κB p65 and MYC in regulating their target genes. Based on the present results, MUC1-C/NF-κB complexes occupy the *NEAT1* gene PLS region and recruit BRG1 and PBRM1 with increases in chromatin accessibility and expression. As confirmation of MUC1-C dependence, silencing MUC1-C decreased occupancy of NF-κB, BRG1, and PBRM1, and chromatin accessibility of *NEAT1* gene PLS region. Silencing MUC1-C similarly decreased occupancy of MYC, BRG1, and PBRM1 on the *NEAT1* dELS region. In concert with these results, MUC1-C, NF-κB, MYC, BRG1, and PBRM1 were necessary for NEAT1_1 and NEAT1_2 expression. **B** MUC1-C/MYC complexes integrate activation of *NEAT1* with regulation of the *SFPQ, NONO* and *FUS* genes. MUC1-C was shown to be necessary for occupancy of MYC, BRG1, and PBRM1 on the (i) *SFPQ* dELS region, and (ii) *NONO* and *FUS* PLS regions. MUC1-C, MYC, BRG1, and PBRM1 were also necessary for chromatin accessibility of the *SFPQ, NONO*, and *FUS* genes and their expression. **C** MUC1-C forms complexes with SFPQ and FUS, but not NONO, and MUC1-C localizes with SFPQ and FUS in paraspeckles. As found for NEAT1_2, SFPQ, NONO, and FUS expression, silencing MUC1-C decreased paraspeckle formation. In terms of functional significance, MUC1-C and NEAT1 drive gene signatures associated with intrinsic chronic inflammation and cancer progression. MUC1-C and NEAT1 were necessary for self-renewal capacity and drug resistance in concert with driving inflammatory memory and the CSC state.

MUC1-C governs auto-inductive circuits that activate inflammatory, proliferative and remodeling pathways associated with the wound-healing response of barrier tissues [14, 15, 42]. These pathways become established by prolonged adaptation of barrier epithelia to chronic inflammation in the progression to cancer [14, 15, 33, 42]. The present results uncover a previously unrecognized role for MUC1-C in integrating activation of the inflammatory NF-κB pathway with driving expression of the *NEAT1* gene in cancer cells. TP53 and other stress-responsive TFs, but not NF-κB to our knowledge, have been linked to *NEAT1* activation [6]. *NEAT1* is widely overexpressed in human cancers and is associated with poor clinical outcomes, which have been attributed in part to an essential role for forming paraspeckles in the response to loss of homeostasis [7]. Our studies demonstrate that MUC1-C/NF-κB complexes occupy a *NEAT1* PLS region. MUC1-C binds directly to NF-κB and promotes NF-κB occupancy on its target genes [14, 15, 42, 44, 54]. Concordant with this function, silencing MUC1-C decreased occupancy of NF-κB on the *NEAT1* PLS region. MUC1-C activates the BAF and PBAF chromatin remodeling complexes and induces global changes in chromatin accessibility across the entire genomes of cancer cells [25–27, 38]. We report that occupancy of MUC1-C/NF-κB complexes on the *NEAT1* PLS recruits BRG1 and PBRM1 in association with increases in chromatin accessibility (Fig. 8A). As confirmation of MUC1-C dependence, targeting MUC1-C decreased chromatin accessibility of the *NEAT1* promoter with downregulation of both NEAT1_1 and NEAT1_2 transcripts [6]. These results identify involvement of MUC1-C in driving NEAT1_1 and NEAT1_2 expression by a mechanism involving chromatin remodeling of the *NEAT1* gene.

The binding of the MUC1-C cytoplasmic domain to the MYC HLH-LZ region regulates expression of MYC target genes [24]. MYC has been linked to induction of NEAT1 expression [6]. Along these lines, we found that MUC1-C is necessary for (i) MYC occupancy of the *NEAT1* PLS region, (ii) recruitment of BRG1 and PBRM1, (iii) PBRM1-driven increases in chromatin accessibility, and (iv) MYC-mediated NEAT1 expression (Fig. 8A). Surprisingly, we found that, in addition to NEAT1, MUC1-C signaling is necessary for induction of the essential SFPQ, NONO and FUS proteins [2, 9], indicating that MUC1-C plays parallel roles in integrating NEAT1 and RBPs necessary for the formation of paraspeckles (Fig. 8B). We found that MUC1-C-induced expression of SFPQ, NONO and FUS is mediated by MYC and not NF-κB. The MUC1-C cytoplasmic domain serine-rich motif confers binding to NF-κB, whereas the redox-sensitive CQC motif binds directly to the MYC HLH-LZ region [15], suggesting that MUC1-C could function in integrating NF-κB- and MYC-mediated induction of NEAT1 with MYC-driven activation of the essential RBP-encoding genes. By way of analogy with *NEAT1* regulation, we found that MUC1-C is necessary for (i) MYC occupancy of the *SFPQ, NONO*, and *FUS* genes, and (ii) PBRM1-dependent increases in chromatin accessibility in association with induction of their expression (Fig. 8B). We also found by coimmunoprecipitation and localization studies that MUC1-C associates with SFPQ and FUS, but not NONO (Fig. 8C). Our results further demonstrate that MUC1-C differentially regulates the abundance of these essential RBPs in that targeting MUC1-C decreased NONO and FUS, while increasing SFPQ levels in the cytoplasm. The functional significance underpinning this differential regulation of NONO and FUS vs SFPQ is unclear; however, SFPQ activates the cytoplasmic RIG-I stress response with increases in IFNβ production [55], which is of interest in that this pathway is induced by MUC1-C in cancer cells in association with chronic inflammation, DNA damage resistance and immune evasion [38, 46]. These results demonstrate that MUC1-C plays a previously unreported role in regulating the expression of SFPQ, NONO, and FUS by a mechanism of chromatin remodeling that is integrated with activation of the *NEAT1* gene.

MUC1-C is activated by disruption of homeostasis and induces lineage plasticity, EMT, and epigenetic reprogramming associated with wound healing [14, 15, 42]. Paraspeckles are increased in response to diverse stress-inducing alterations, which include inflammation, cell lineage transitions, and cancer [10, 56, 57]. MUC1 first appeared in mammals and, along these lines, paraspeckles are mammalian-specific structures [14, 15, 42, 58]. The evolutionary concordance of MUC1-C and paraspeckles supported the contention that they may intersect in promoting the response to loss of homeostasis, which in settings of chronic inflammation could contribute to cancer (Fig. 8C). Underpinning this intersection, dysregulation of MUC1-C [14, 15, 42] and paraspeckle formation [50–52, 57, 59] each contribute to cancer progression. In line with their integration in promoting cancer, our results further demonstrate that MUC1-C and NEAT1 regulate common sets of genes associated with chronic inflammation, induction of EMT and activation of the NOTCH1/2 stemness factors. The CSC state is of importance in conferring therapeutic resistance [60–63]. We found that MUC1-C and NEAT1 contribute to cytotoxic drug resistance in different cancer models. In addition, our results consolidate the interaction between MUC1-C and NEAT1 by demonstrating in enriched CSC populations that (i) MUC1-C is necessary for regulation of NEAT1_2, and (ii) targeting NEAT1 suppresses MUC1-C expression. To lend further credence to the MUC1-C/NEAT1 auto-regulatory pathway, we show that MUC1-C and NEAT1 are necessary for CSC self-renewal capacity. These results collectively indicate that (i) MUC1-C is required for NEAT1 expression and thereby paraspeckle formation and (ii) MUC1-C and NEAT1 contribute to the regulation of genes that promote cancer progression. In this way, MUC1-C integrates changes in chromatin with the formation of subnuclear paraspeckles to regulate gene expression.

Our results further indicate that MUC1-C could contribute to the formation of other biomolecular condensates. Along these lines, MUC1-C (i) regulates SFPQ, NONO, and FUS expression, and (ii) forms complexes with SFPQ and FUS that promote LLPS in other settings, such as at sites of DNA damage repair [64–66]. MUC1-C also localizes to PML bodies that, like paraspeckles, are biomolecular condensates restricted to mammals [67]. Additionally, MUC1-C regulates (i) the XIST lncRNA [68], which initiates X chromosome inactivation by forming phase-separated structures [69, 70], and (ii) TDP-43 [68], which regulates XIST and paraspeckle formation [71]. MUC1-C is modified by galectin-3, includes an intrinsically disordered domain, forms multimers, and binds to RNPs, which are characteristics that can contribute to the formation of biomolecular condensates [15, 42, 72]. Subsequent studies will therefore be needed to determine if MUC1-C is necessary for the formation of other nuclear or cytoplasmic stress bodies.

## Materials and methods

### Cell culture

Human BT-549 TNBC cells (ATCC, Manassas, VA, USA) were cultured in RPMI1640 medium (Thermo Fisher Scientific, Waltham, MA, USA) with 10% fetal bovine serum (FBS; GEMINI Bio-Products, West Sacramento, CA, USA), 100 μg/ml streptomycin, 100 U/ml penicillin and 10 μg/ml insulin. Human MDA-MB-436 TNBC cells (ATCC) were cultured in RPMI1640 medium (Thermo Fisher Scientific, Waltham, MA, USA) with 10% fetal bovine serum (FBS; GEMINI Bio-Products, West Sacramento, CA, USA), 100 μg/ml streptomycin and 100 U/ml penicillin. Human MDA-MB-468 TNBC cells (ATCC) were cultured in Leibovitz’s L-15 medium (Thermo Fisher Scientific) containing 10% FBS. DU-145 CRPC cells (ATCC) were cultured in RPMI1640 medium (Corning Life Sciences, Corning, NY, USA) with 10% heat-inactivated FBS. Cells were treated with the NF-κB inhibitor BAY11-7082 (S2913; Selleckchem, Houston, TX, USA) and MUC1-C inhibitor GO-203.

Short tandem repeat (STR) analysis was performed for authentication of the cells. Mycoplasma contamination was monitored using the MycoAlert Mycoplasma Detection Kit (Lonza, Rockland, MA, USA). Cells were cultured for 3 months when performing experiments.

### Gene silencing and rescue

MUC1shRNA (MISSION shRNA TRCN0000122938; Sigma, St. Louis, MO, USA), MYCshRNA (MISSION shRNA TRCN0000039642; Sigma) or a control scrambled CshRNA (Sigma) was integrated into pLKO-tet-puro (Plasmid #21915; Addgene, Cambridge, MA, USA) as described [31]. The MUC1shRNA#2 (MISSION shRNA TRCN0000430218), PBRM1shRNA (MISSION shRNA TRCN0000235890), NF-κBshRNA (MISSION shRNA TRCN0000014687), BRG1shRNA (MISSION shRNA TRCN0000231102) and ARID1AshRNA (MISSION shRNA TRCN0000059090) was produced in HEK293T cells as described [46]. shRNA targeting NEAT1 (5’-CATGGACCGTGGTTTGTTACT3’) was purchased from GenePharma (Shanghai, China). MUC1-C cDNA and Flag-tagged MUC1-CD [73] were inserted into pInducer20 (Plasmid #44012, Addgene) [74]. Transduced cells were selected for growth in 1–2 μg/ml puromycin. Cells were cultured in 0.1% DMSO as the vehicle control or 500 ng/ml DOX (Millipore Sigma). Control siRNA (Cat no-4390843) and NEAT1 targeted siRNA (Cat. no-4390771, ID-n272457) (ThermoFisher Scientific) were transfected into cells using Lipofectamine 3000 (Invitrogen).

### Quantitative reverse-transcription PCR (qRT-PCR)

Total cellular RNA was isolated with Trizol reagent (Thermo Fisher Scientific). The High Capacity cDNA Reverse-Transcription Kit (Applied Biosystems, Grand Island, NY, USA) was used for cDNA synthesis) as described [31]. cDNAs were amplified with the Power SYBR Green PCR Master Mix (Applied Biosystems) and the CFX96 Real-Time PCR System (BIO-RAD, Hercules, CA, USA) as described [31].

### Click-iT Nascent RNA Assay

Nascent RNA labeling with EU was performed using the Click-iT Nascent RNA Capture kit (Invitrogen) according to the manufacturer’s protocol. Briefly, cells were pulsed with 0.5 mM EU for 24 h. Total RNA was then isolated, and the nascent transcripts were captured on streptavidin magnetic beads. cDNA synthesis was performed using the Superscript VILO cDNA synthesis kit (Invitrogen) followed by analysis with qRT-PCR.

### Immunoblot analysis

Total lysates prepared from non-confluent cells were subjected to immunoblot analysis using anti-MUC1-C (HM-1630-P1ABX, 1:1000 dilution; Thermo Fisher Scientific), anti-β-actin (A5441, 1:5000 dilution; Sigma-Aldrich), anti-GAPDH (5174, 1:5000 dilution; CST), anti-SFPQ (ab177149, 1:1000 dilution; Abcam), anti-NONO (#90336, 1:1000 dilution, CST), anti-FUS (ab124923, 1:1000 dilution, Abcam), anti-total H3 (ab18521, 1:1000 dilution, Abcam), anti-MYC (#ab32072, 1:1000 dilution; Abcam), anti-tubulin (#2144S, 1:1000 dilution; CST),anti-NOTCH1 (#3608, 1:1000 dilution; CST), anti-NOTCH2 (#5732, 1:1000 dilution; CST) and anti-HEY1 (#19929-1-AP,1:1000 dilution; Proteintech).

### Chromatin immunoprecipitation studies

Cells were crosslinked with 1% formaldehyde for 5 min at 37 °C, quenched with 2 M glycine, washed with PBS, and then sonicated in a Covaris E220 sonicator to generate 300–600 bp DNA fragments. Immunoprecipitations were performed with a control IgG (3900S, CST) or antibodies against MUC1-C (#16564S, CST), MYC (#ab32072; Abcam), NF-κB p65 (#ab16502, Abcam), BRG1 (#ab8580, Abcam) and anti-PBRM1 (A301-591A; Bethyl Laboratories). Immunoprecipitated DNA was analyzed using SYBR-green and the CFX384 real-time PCR machine (Bio-Rad, USA). Data are reported as a percentage of input DNA for each sample.

### RNA-seq analysis

Total RNA was isolated with the RNeasy Plus Mini Kit (Qiagen) from cells cultured in triplicates. Library preparation was performed using TruSeq Stranded mRNA (Illumina, San Diego, CA, USA) as described [28]. Human genome (GRCh38.74) was used to align raw sequencing reads with STAR as described [28]. Gene counts were normalized and differential expression analysis was performed using DESeq2 as described [28]. The fgsea (v1.8.0) package in R was used for differential expression rank order and GSEA. Queried gene sets included those available through the Molecular Signatures Database (MsigDB) as described [28].

### Coimmunoprecipitation studies

Protein lysates were immunoprecipitated with anti-MUC1-C (HM-1630-P1ABX; Thermo Fisher Scientific) or a rabbit isotype control IgG (3900S, CST) using the Pierce Classic Magnetic Co-IP Kit (Thermo Fisher Scientific).

### Cell proliferation assays

BT-549/PTX-R cells and DU-145/DTX-R cells with and without NEAT1 silencing were seeded at 6000 cells per well in 96-well plates. After 24 h, cells were treated with 1 nM PTX for 24, 48, and 72 h or 1 nM Docetaxel for 24,48 and 72 h. The Alamar Blue assay was used to assess cell viability (Thermo Fisher Scientific). Fluorescence intensity (560 nm excitation/590 nm emission) was measured in quintuplicate.

### Colony formation assays

Cells were seeded in 24-well plates. After 7–10 days, cells were stained with 0.5% crystal violet/25% methanol (LabChem, Zelienople, PA, USA). Colonies were counted in triplicate wells.

### Tumorsphere formation assays

Single-cell suspensions were cultured in MammoCult Human MediumKit (Stemcell Technologies) at a density of 5000 cells/well of a 6-well ultralow attachment culture plate (Corning) for 10 days as described [27]. Tumorspheres with a diameter >50 microns were counted under an inverted microscope in triplicate wells.

### RNAscope and protein multiplexing

Cells were grown on chambered slides, fixed with freshly made 4% PFA for 10 min, washed with 1X PBS and permeabilized with PBS-Triton (0.5%) for 15 min at room temperature. Cells were washed with PBS-Triton (0.1%) and then twice in 2× SSC-T for 5 min at room temperature at each wash step. The slides were rinsed twice in MilliQ-grade water and treated with the ACDBio Hydrogen Peroxide pre-treatment for 10 min at room temperature. The slides were then rinsed with MilliQ-grade water and the cells were incubated with RNAScope probes against (i) NEAT1 (1125421-C1; bio-techne, Minneapolis, MN, USA) overnight at 40 °C. Probe signal amplification was performed using the ACDBio RNAScope 2.5 HD Detection Brown kit (322310; bio-techne) and reaction with Biotium CF647 Tyramide (96022; bio-techne) to visualize probe staining. Slides were washed with TBS-T and then incubated with primary antibodies against MUC1-C (HM-1630-P1ABX; Thermo Fisher Scientific) overnight at 4 °C. After washing again with TBS-T, slides were incubated with AF568-tagged secondary antibodies against mouse IgG (A10037; Thermo Fisher Scientific) and Armenian hamster AF488-tagged Ig (ab173003; Abcam) for 2 h at room temperature. Slides were washed with TBS-T, stained with DAPI, mounted with Prolong Gold and visualized under a confocal microscope.

### Immunofluorescence analysis

BT-549 cells were fixed in 4% paraformaldehyde (Sigma) at room temperature for 10 min. Samples were treated with 0.1% Triton X-100 (Sigma) at room temperature for 10 min, blocked with 3% Normal Goat Serum (Gibco), incubated with anti-SFPQ (ab177149, 1:200 dilution; Abcam), anti-NONO (#90336, 1:200 dilution, CST), anti-FUS (ab124923, 1:200 dilution, Abcam), and anti-PSPC1 (no-1671-41-AP, 1:100 dilution, Proteintech) at 4°C overnight and then incubated with goat anti-rabbit IgG H and L labeled with Alexa Fluor 488 (Abcam), anti-hamster IgG Alexa Fluor 568(Abcam) at room temperature for 1 h. Invitrogen™ ProLong™ Diamond Antifade Mountant with DAPI (Invitrogen) was used for staining of nuclei. The cells were analyzed using a Zeiss 980 Confocal microscope. Colocalization analysis was performed by (i) Zeiss’s Zen software and (ii) ImageJ plugin JACoP (Just Another Colocalization Plugin; https://github.com/fabricecordelieres/IJ-Plugin_JACoP/releases). Pearson’s coefficient was used as a colocalization indicator. The number of speckles were quantified using Cellpose in Cell Profiler and ImageJ software.

### ATAC-seq

ATAC-seq libraries were generated from three biologically independent replicates per condition as described [27, 75]. The raw ATAC-seq data were processed using the pipeline: (https://github.com/macs3-project/genomics-analysis-pipelines). To generate the signal tracks for the Integrative Genome Browser (IGV) snapshots, we used MACS2 to pileup the aligned ATAC-seq read pairs and normalized the pileup values by the million read depth of each library as described [27].

### Chromatin accessibility assay

DNAse1 chromatin accessibility assays were performed on chromatin isolated as described [27]. Aliquots of chromatin were left untreated or digested with 3 U/100 μl Dnase I (Promega, Madison, WI, USA) for 5 min at room temperature as described [27]. DNA was purified and amplified by qPCR using primers listed in Supplementary Table S2. qPCR results were analyzed according to the formula 100/2^Ct (Dnase I) −Ct (no Dnase I)^. The data were normalized to input DNA without Dnase I treatment.

### Flow cytometry

Cells were washed with PBS and harvested with 0.05% TryplE. Cells were resuspended in FACS staining buffer (Invitrogen) (10^6^ cells/100 μl). Conjugated anti-human CD44 (FITC, cat. #555478; BD Biosciences, San Diego, CA, USA) was added to the cell suspension at concentrations recommended by the manufacturer and then incubated at 4 °C in the dark for 30–40 min. The labeled cells were washed in the staining buffer, then fixed in PBS containing 2% paraformaldehyde, and then analyzed on MACSQuant Analyzer 10 Flow Cytometer (Miltenyi Biotec, Waltham, MA) to acquire 20,000 events for each sample. Data were analyzed with FlowJo v10.6.2 (BD Biosciences) software.

### Analysis of human TNBC tumors

Raw Read Counts of the Breast Invasive Carcinoma data collection were downloaded from TCGA using TCGAbiolinks (version 2.30). Reads were normalized to TMM (Trimmed Mean of M-values) values using endeR for subsequent Spearman’s Correlation Analysis. Survival curves based on MUC1 and NEAT1 expression levels were generated using the Kaplan–Meier Plotter (http://kmplot.com/analysis/). Patients with grade 3 breast cancer treated with chemotherapy were included in this analysis. The statistical difference was calculated using the log-rank test. The prognostic value of gene expression levels was assessed using the Cox proportional hazards regression model.

### Statistics

Each experiment was performed at least three times. Data are expressed as the mean ± SD. The unpaired Mann–Whitney *U*-test was used to determine differences between means of groups. A *p* value of <0.05 denoted by an asterisk (*) was considered statistically significant.

### Supplementary information

Supplementary data

## Data Availability

The accession numbers for the (i) RNA-seq data are GEO Submission GSE164141 and GSE247441, and (ii) ATAC-seq data are GEO Submission GSE180599.

## References

[1] Fox AH, Nakagawa S, Hirose T, Bond CS (2018). Paraspeckles: where long noncoding RNA meets phase separation. Trends Biochem Sci.

[2] Naganuma T, Nakagawa S, Tanigawa A, Sasaki YF, Goshima N, Hirose T (2012). Alternative 3'-end processing of long noncoding RNA initiates construction of nuclear paraspeckles. EMBO J.

[3] Wilusz JE, JnBaptiste CK, Lu LY, Kuhn CD, Joshua-Tor L, Sharp PA (2012). A triple helix stabilizes the 3' ends of long noncoding RNAs that lack poly(A) tails. Genes Dev.

[4] Wang Y, Hu SB, Wang MR, Yao RW, Wu D, Yang L (2018). Genome-wide screening of NEAT1 regulators reveals cross-regulation between paraspeckles and mitochondria. Nat Cell Biol.

[5] Wang Z, Li K, Huang W (2020). Long non-coding RNA NEAT1-centric gene regulation. Cell Mol Life Sci.

[6] Wang Y, Chen LL (2020). Organization and function of paraspeckles. Essays Biochem.

[7] Smith NE, Spencer-Merris P, Fox AH, Petersen J, Michael MZ (2022). The long and the short of it: NEAT1 and cancer cell metabolism. Cancers.

[8] Hirose T, Virnicchi G, Tanigawa A, Naganuma T, Li R, Kimura H (2014). NEAT1 long noncoding RNA regulates transcription via protein sequestration within subnuclear bodies. Mol Biol Cell.

[9] Kawaguchi T, Tanigawa A, Naganuma T, Ohkawa Y, Souquere S, Pierron G (2015). SWI/SNF chromatin-remodeling complexes function in noncoding RNA-dependent assembly of nuclear bodies. Proc Natl Acad Sci USA.

[10] McCluggage F, Fox AH (2021). Paraspeckle nuclear condensates: global sensors of cell stress?. Bioessays.

[11] Laurenzi T, Palazzolo L, Taiana E, Saporiti S, Ben Mariem O, Guerrini U (2022). Molecular modelling of NONO and SFPQ dimerization process and RNA recognition mechanism. Int J Mol Sci.

[12] Yamazaki T, Souquere S, Chujo T, Kobelke S, Chong YS, Fox AH (2018). Functional domains of NEAT1 architectural lncRNA Induce paraspeckle assembly through phase separation. Mol Cell.

[13] Kufe D (2009). Mucins in cancer: function, prognosis and therapy. Nat Rev Cancer.

[14] Kufe D (2020). MUC1-C in chronic inflammation and carcinogenesis; emergence as a target for cancer treatment. Carcinogenesis.

[15] Kufe D (2022). Emergence of MUC1 in mammals for adaptation of barrier epithelia. Cancers.

[16] Alam M, Bouillez A, Tagde A, Ahmad R, Rajabi H, Maeda T (2016). MUC1-C represses the Crumbs complex polarity factor CRB3 and downregulates the Hippo pathway. Mol Cancer Res.

[17] Rajabi H, Alam M, Takahashi H, Kharbanda A, Guha M, Ahmad R (2014). MUC1-C oncoprotein activates the ZEB1/miR-200c regulatory loop and epithelial-mesenchymal transition. Oncogene.

[18] Hata T, Rajabi H, Yamamoto M, Jin C, Ahmad R, Zhang Y (2019). Targeting MUC1-C inhibits TWIST1 signaling in triple-negative breast cancer. Mol Cancer Ther.

[19] Hiraki M, Maeda T, Bouillez A, Alam M, Tagde A, Hinohara K (2017). MUC1-C activates BMI1 in human cancer cells. Oncogene.

[20] Rajabi H, Hiraki M, Tagde A, Alam M, Bouillez A, Christensen CL (2017). MUC1-C activates EZH2 expression and function in human cancer cells. Sci Rep.

[21] Rajabi H, Tagde A, Alam M, Bouillez A, Pitroda S, Suzuki Y (2016). DNA methylation by DNMT1 and DNMT3b methyltransferases is driven by the MUC1-C oncoprotein in human carcinoma cells. Oncogene.

[22] Rajabi H, Hiraki M, Kufe D (2018). MUC1-C activates polycomb repressive complexes and downregulates tumor suppressor genes in human cancer cells. Oncogene.

[23] Bhattacharya A, Fushimi A, Wang K, Yamashita N, Morimoto Y, Ishikawa S (2023). MUC1-C intersects chronic inflammation with epigenetic reprogramming by regulating the SET1A compass complex in cancer progression. Comms Biol.

[24] Hata T, Rajabi H, Takahashi H, Yasumizu Y, Li W, Jin C (2019). MUC1-C activates the NuRD complex to drive dedifferentiation of triple-negative breast cancer cells. Cancer Res.

[25] Hagiwara M, Yasumizu Y, Yamashita N, Rajabi H, Fushimi A, Long MD (2021). MUC1-C activates the BAF (mSWI/SNF) complex in prostate cancer stem cells. Cancer Res.

[26] Hagiwara M, Fushimi A, Yamashita N, Battacharya A, Rajabi H, Long M (2021). MUC1-C activates the PBAF chromatin remodeling complex in integrating redox balance with progression of human prostate cancer stem cells. Oncogene.

[27] Bhattacharya A, Fushimi A, Yamashita N, Hagiwara M, Morimoto Y, Rajabi H (2022). MUC1-C dictates JUN and BAF-mediated chromatin remodeling at enhancer signatures in cancer stem cells. Mol Cancer Res.

[28] Yasumizu Y, Rajabi H, Jin C, Hata T, Pitroda S, Long MD (2020). MUC1-C regulates lineage plasticity driving progression to neuroendocrine prostate cancer. Nat Commun.

[29] Li W, Zhang N, Jin C, Long MD, Rajabi H, Yasumizu Y (2020). MUC1-C drives stemness in progression of colitis to colorectal cancer. JCI Insight.

[30] Luan Z, Morimoto Y, Fushimi A, Yamashita N, Suo W, Bhattacharya A (2021). MUC1-C dictates neuroendocrine lineage specification in pancreatic ductal adenocarcinomas. Carcinogenesis.

[31] Fushimi A, Morimoto Y, Ishikawa S, Yamashita N, Bhattacharya A, Daimon T (2022). Dependence on the MUC1-C oncoprotein in classic, variant and non-neuroendocrine small cell lung cancer. Mol Cancer Res.

[32] Morimoto Y, Fushimi A, Yamashita N, Hagiwara M, Bhattacharya A, Cheng J (2022). Addiction of Merkel cell carcinoma to MUC1-C identifies a potential new target for treatment. Oncogene.

[33] Yamashita N, Kufe D (2022). Addiction of cancer stem cells to MUC1-C in triple-negative breast cancer progression. Int J Mol Sci.

[34] Yamamoto M, Jin C, Hata T, Yasumizu Y, Zhang Y, Hong D (2019). MUC1-C integrates chromatin remodeling and PARP1 activity in the DNA damage response of triple-negative breast cancer cells. Cancer Res.

[35] Shigeta K, Hasegawa M, Kikuchi E, Yasumizu Y, Kosaka T, Mizuno R (2020). Role of the MUC1-C oncoprotein in the acquisition of cisplatin resistance by urothelial carcinoma. Cancer Sci.

[36] Yamashita N, Long M, Fushimi A, Yamamoto M, Hata T, Hagiwara M (2021). MUC1-C integrates activation of the IFN-gamma pathway with suppression of the tumor immune microenvironment in triple-negative breast cancer. J Immunother Cancer.

[37] Hagiwara M, Fushimi A, Bhattacharya A, Yamashita N, Morimoto Y, Oya M (2022). MUC1-C integrates type II interferon and chromatin remodeling pathways in immunosuppression of prostate cancer. OncoImmunol.

[38] Yamashita N, Morimoto Y, Fushimi A, Ahmad R, Bhattacharya A, Daimon T (2023). MUC1-C dictates PBRM1-mediated chronic induction of interferon signaling, DNA damage resistance and immunosuppression in triple-negative breast cancer. Mol Cancer Res.

[39] Li W, Han Y, Sun C, Li X, Zheng J, Che J (2022). Novel insights into the roles and therapeutic implications of MUC1 oncoprotein via regulating proteins and non-coding RNAs in cancer. Theranostics.

[40] Zhang Y, Yang M, Yang S, Hong F (2022). Role of noncoding RNAs and untranslated regions in cancer: a review. Med (Baltim).

[41] Leng Y, Cao C, Ren J, Huang L, Chen D, Ito M (2007). Nuclear import of the MUC1-C oncoprotein is mediated by nucleoporin Nup62. J Biol Chem.

[42] Kufe D (2022). Chronic activation of MUC1-C in wound repair promotes progression to cancer stem cells. J Cancer Metastasis Treat.

[43] Zhou W, Chen X, Hu Q, Chen X, Chen Y, Huang L (2018). Galectin-3 activates TLR4/NF-kappaB signaling to promote lung adenocarcinoma cell proliferation through activating lncRNA-NEAT1 expression. BMC Cancer.

[44] Ahmad R, Raina D, Joshi MD, Kawano T, Kharbanda S, Kufe D (2009). MUC1-C oncoprotein functions as a direct activator of the NF-kappaB p65 transcription factor. Cancer Res.

[45] Marshall AC, Cummins J, Kobelke S, Zhu T, Widagdo J, Anggono V (2023). Different low-complexity regions of SFPQ play distinct roles in the formation of biomolecular condensates. J Mol Biol.

[46] Yamashita N, Fushimi A, Morimoto Y, Bhattacharya A, Hagiwara M, Yamamoto M (2022). Targeting MUC1-C suppresses chronic activation of cytosolic nucleotide receptors and STING in triple-negative breast cancer. Cancers.

[47] Yamashita N, Withers H, Morimoto Y, Bhattacharya A, Haratake N, Diamon T (2023). MUC1-C integrates aerobic glycolysis with suppression of oxidative phosphorylation in triple-negative breast cancer stem cells. iScience.

[48] Lan Q, Liu PY, Haase J, Bell JL, Huttelmaier S, Liu T (2019). The critical role of RNA m(6)A methylation in cancer. Cancer Res.

[49] Pan Y, Ma P, Liu Y, Li W, Shu Y (2018). Multiple functions of m(6)A RNA methylation in cancer. J Hematol Oncol.

[50] Dong P, Xiong Y, Yue J, Hanley SJB, Kobayashi N, Todo Y (2018). Long non-coding RNA NEAT1: a novel target for diagnosis and therapy in human tumors. Front Genet.

[51] Klec C, Prinz F, Pichler M (2019). Involvement of the long noncoding RNA NEAT1 in carcinogenesis. Mol Oncol.

[52] Shin VY, Chen J, Cheuk IW, Siu MT, Ho CW, Wang X (2019). Long non-coding RNA NEAT1 confers oncogenic role in triple-negative breast cancer through modulating chemoresistance and cancer stemness. Cell Death Dis.

[53] Ben-Porath I, Thomson MW, Carey VJ, Ge R, Bell GW, Regev A (2008). An embryonic stem cell-like gene expression signature in poorly differentiated aggressive human tumors. Nat Genet.

[54] Ahmad R, Raina D, Trivedi V, Ren J, Rajabi H, Kharbanda S (2007). MUC1 oncoprotein activates the IκB kinase β complex and constitutive NF-κB signaling. Nat Cell Biol.

[55] Ma H, Han P, Ye W, Chen H, Zheng X, Cheng L (2017). The long noncoding RNA NEAT1 exerts antihantaviral effects by acting as positive feedback for RIG-I signaling. J Virol.

[56] Pan Y, Wang T, Zhao Z, Wei W, Yang X, Wang X (2022). Novel insights into the emerging role of Neat1 and its effects downstream in the regulation of inflammation. J Inflamm Res.

[57] Adriaens C, Standaert L, Barra J, Latil M, Verfaillie A, Kalev P (2016). p53 induces formation of NEAT1 lncRNA-containing paraspeckles that modulate replication stress response and chemosensitivity. Nat Med.

[58] Rasko T, Pande A, Radscheit K, Zink A, Singh M, Sommer C (2022). A novel gene controls a new structure: piggyBac transposable element-derived 1, umammal-specific neuronal paraspeckles. Mol Biol Evol.

[59] Gu J, Zhang B, An R, Qian W, Han L, Duan W (2022). Molecular interactions of the long noncoding RNA NEAT1 in cancer. Cancers.

[60] De Angelis ML, Francescangeli F, La Torre F, Zeuner A (2019). Stem cell plasticity and dormancy in the development of cancer therapy resistance. Front Oncol.

[61] Miranda A, Hamilton PT, Zhang AW, Pattnaik S, Becht E, Mezheyeuski A (2019). Cancer stemness, intratumoral heterogeneity, and immune response across cancers. Proc Natl Acad Sci USA.

[62] Malta TM, Sokolov A, Gentles AJ, Burzykowski T, Poisson L, Weinstein JN (2018). Machine learning identifies stemness features associated with oncogenic dedifferentiation. Cell.

[63] Quintanal-Villalonga A, Chan JM, Yu HA, Pe’er D, Sawyers CL, Sen T (2020). Lineage plasticity in cancer: a shared pathway of therapeutic resistance. Nat Rev Clin Oncol.

[64] Murray DT, Kato M, Lin Y, Thurber KR, Hung I, McKnight SL (2017). Structure of FUS protein fibrils and its relevance to self-assembly and phase separation of low-complexity domains. Cell.

[65] Levone BR, Lenzken SC, Antonaci M, Maiser A, Rapp A, Conte F (2021). FUS-dependent liquid-liquid phase separation is important for DNA repair initiation. J Cell Biol.

[66] Yasuhara T, Xing YH, Bauer NC, Lee L, Dong R, Yadav T (2022). Condensates induced by transcription inhibition localize active chromatin to nucleoli. Mol Cell.

[67] Lacroix E, Audas TE (2022). Keeping up with the condensates: the retention, gain, and loss of nuclear membrane-less organelles. Front Mol Biosci.

[68] Wang K, Bhattacharya A., Haratake N, et al. XIST and MUC1-C form an auto-regulatory pathway in driving cancer progression. Cell Death Dis. 2024;15:330. 10.1038/s41419-024-06684-9 PMC11091074 38740827

[69] Cerase A, Armaos A, Neumayer C, Avner P, Guttman M, Tartaglia GG (2019). Phase separation drives X-chromosome inactivation: a hypothesis. Nat Struct Mol Biol.

[70] Quinodoz SA, Jachowicz JW, Bhat P, Ollikainen N, Banerjee AK, Goronzy IN (2021). RNA promotes the formation of spatial compartments in the nucleus. Cell.

[71] Modic M, Grosch M, Rot G, Schirge S, Lepko T, Yamazaki T (2019). Cross-regulation between TDP-43 and paraspeckles promotes pluripotency-differentiation transition. Mol Cell.

[72] Voss PG, Wang JL (2023). Liquid-liquid phase separation: galectin-3 in nuclear speckles and ribonucleoprotein complexes. Exp Cell Res.

[73] Huang L, Liao X, Beckett M, Li Y, Khanna KK, Wang Z (2010). MUC1-C oncoprotein interacts directly with ATM and promotes the DNA damage response to ionizing radiation. Genes Cancer.

[74] Meerbrey KL, Hu G, Kessler JD, Roarty K, Li MZ, Fang JE (2011). The pINDUCER lentiviral toolkit for inducible RNA interference in vitro and in vivo. Proc Natl Acad Sci USA.

[75] Buenrostro JD, Wu B, Chang HY, Greenleaf WJ (2015). ATAC-seq: a method for assaying chromatin accessibility genome-wide. Curr Protoc Mol Biol.

