# Supplementary material for: MUC1-C regulates NEAT1 lncRNA expression and paraspeckle formation in cancer progression
Source: Oncogene. 2024 May 27;43(28):2199–214. doi: 10.1038/s41388-024-03068-3 (PMC11226401; doi:10.1038/s41388-024-03068-3)
Supplement: Supplementary file 1 — Supplementary data [file 41388_2024_3068_MOESM1_ESM.pdf]

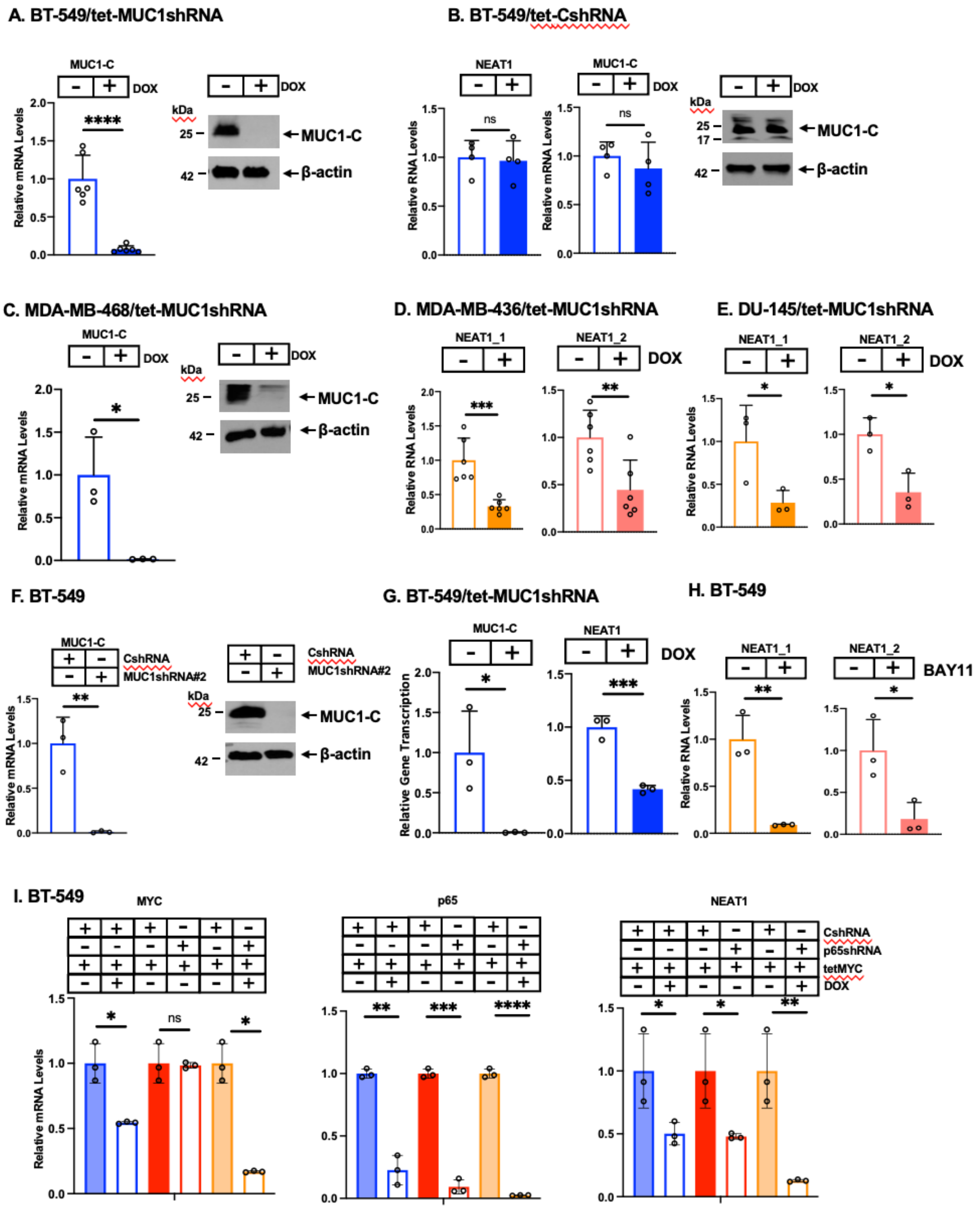


**Supplemental Figure S1. Effects of silencing MUC1-C on NEAT1_1 and NEAT1_2 expression. A.** BT-549/tet-MUC1shRNA cells treated with vehicle or DOX for 7 days were analyzed for MUC1-C expression by qRT-PCR using primers listed in Supplemental Table S1. The results (mean±SD of 3 replicates) are expressed as relative mRNA levels compared to that obtained for vehicle-treated cells (assigned a value of 1) (left). Lysates were immunoblotted with antibodies against the indicated proteins (right). **B.** BT-549/tet-CshRNA cells treated with vehicle or DOX for 7 days were analyzed for NEAT1 and MUC1-C expression by qRT-PCR. The results (mean±SD of 3 replicates) are expressed as relative RNA levels compared to that obtained for vehicle-treated cells (assigned a value of 1) (left). Lysates were immunoblotted with antibodies against the

indicated proteins (right). **C.** MDA-MB-468/tet-MUC1shRNA cells treated with vehicle or DOX for 7 days were analyzed for MUC1-C expression by qRT-PCR. The results (mean±SD of 3 replicates) are expressed as relative mRNA levels compared to that obtained for vehicle-treated cells (assigned a value of 1) (left). Lysates were immunoblotted with antibodies against the indicated proteins (right). **D and E.** MDA-MB-436/tet-MUC1shRNA (**D**) and DU-145/tet-MUC1shRNA (**E**) cells treated with vehicle or DOX for 7 days were analyzed for NEAT1_1 and NEAT1_2 transcripts by qRT-PCR. The results (mean±SD of 3 biologic replicates) are expressed as relative levels compared to that obtained for vehicle-treated cells (assigned a value of 1). **F.** BT-549/CshRNA and BT-549/MUC1shRNA#2 cells were analyzed for MUC1-C transcripts by qRT-PCR. The results (mean±SD of 3 replicates) are expressed as relative levels compared to that obtained for CshRNA-expressing cells (assigned a value of 1) (left). Lysates were immunoblotted with antibodies against the indicated proteins (right). **G.** BT-549/tet-MUC1shRNA cells treated with vehicle or DOX for 7 days were analyzed for nascent MUC1-C and NEAT1 transcription. The results (mean±SD of 3 replicates) are expressed as relative transcription levels compared to that obtained in vehicle-treated cells (assigned a value of 1). **H.** BT-549 cells treated with vehicle or 5 μM BAY11 for 48 hours were analyzed for NEAT1_1 and NEAT1_2 transcripts by qRT-PCR. The results (mean±SD of 3 determinations) are expressed as relative RNA levels compared to that obtained for vehicle-treated cells (assigned a value of 1). **I.** BT-549 cells expressing the indicated vectors were analyzed for MYC, NF-κB p65 and NEAT1 transcripts by qRT-PCR. The results (mean±SD of 3 determinations) are expressed as relative RNA levels compared to that obtained for control cells (assigned a value of 1).

Supplemental 2

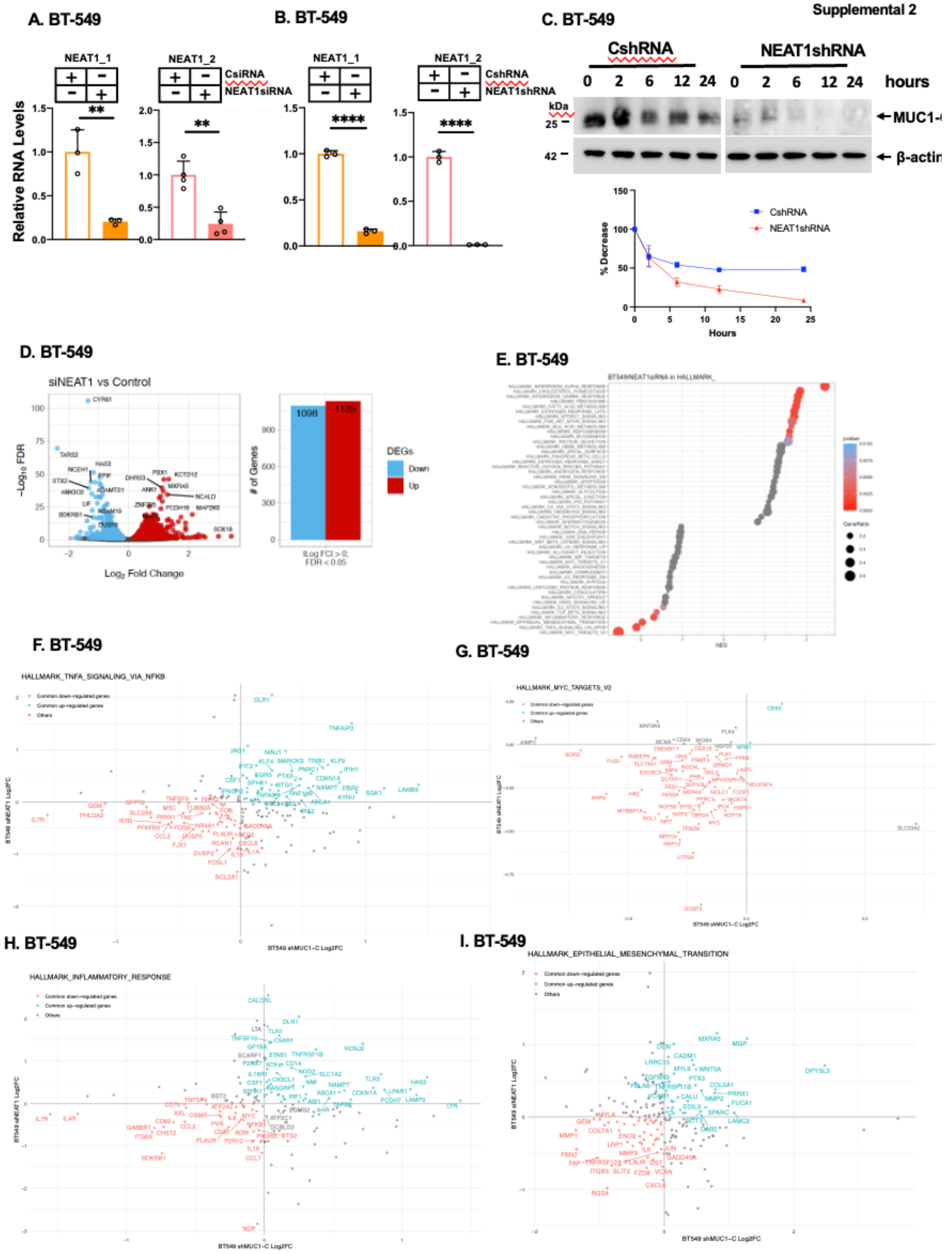


**Supplemental Figure S2. MUC1-C and NEAT1 regulate common sets of genes associated with chronic inflammation and EMT. A and B.** BT-549/CsiRNA and BT-549/NEAT1siRNA (**A**) and BT-549/CshRNA and BT-549/NEAT1shRNA (**B**) cells were analyzed for NEAT1_1 and NEAT1_2

transcripts by qRT-PCR. The results (mean±SD of 3 biologic replicates) are expressed as relative RNA levels compared to that obtained for CsiRNA/CshRNA cells (assigned a value of 1). **C.** BT-549/CshRNA and BT-549/NEAT1shRNA cells were treated with 50 ug/ml cycloheximide (CHX) for the indicated times. Lysates were immunoblotted with antibodies against the indicated proteins. **D.** Volcano plots of downregulated and upregulated genes in BT-549/NEAT1siRNA vs BT-549/CsiRNA cells. **E.** Analysis of 549/NEAT1siRNA vs BT-549/CsiRNA RNA-seq data using the indicated HALLMARK gene signatures. **F-I.** Comparisons of downregulated and upregulated genes in NEAT1- and MUC1-C-silenced cells using the indicated gene signatures.

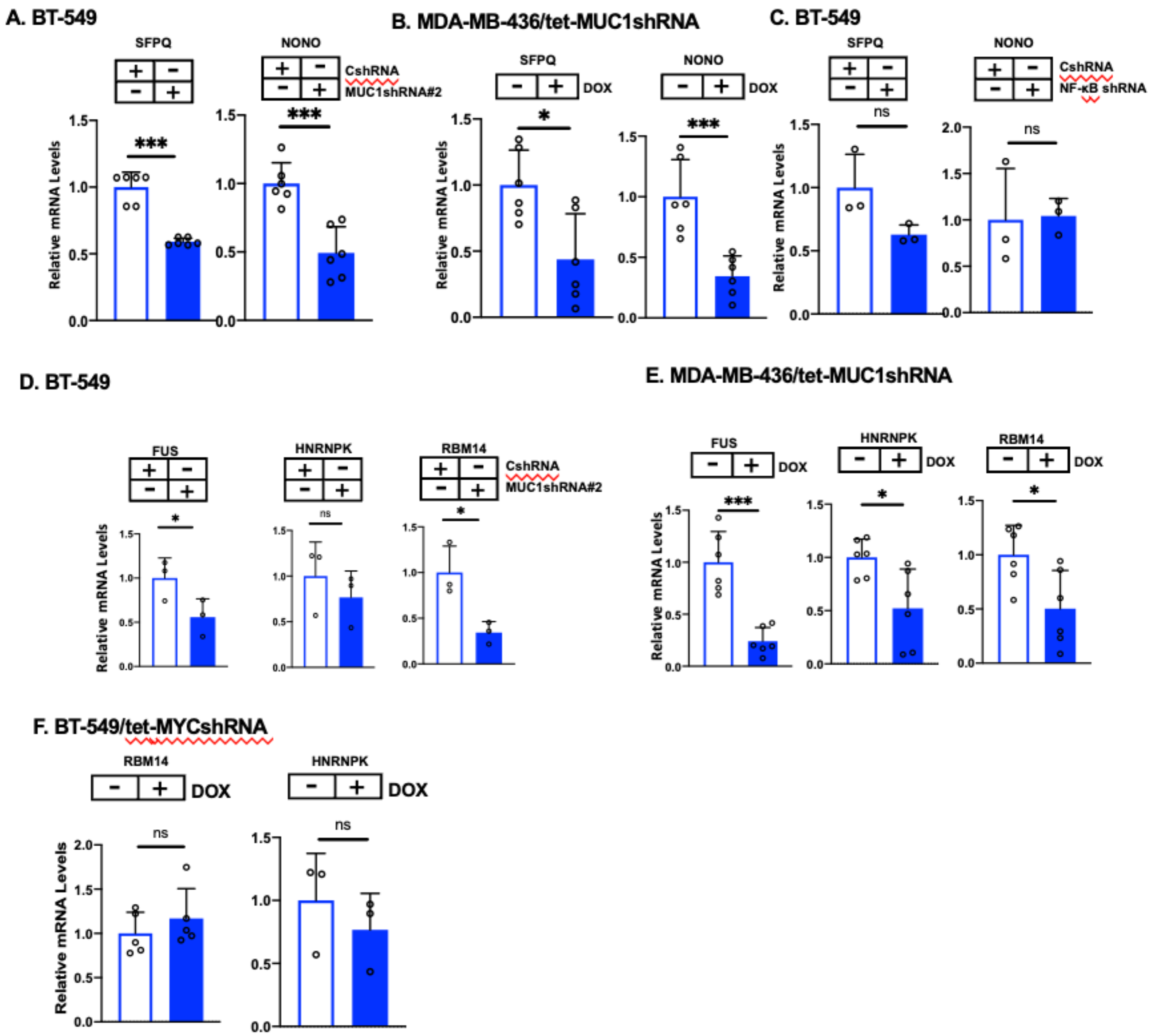


**Supplemental Figure S3. Effects of silencing MUC1-C, NF-κB and MYC on RBP expression. A.** BT-549/CshRNA and BT-549/MUC1shRNA#2 cells were analyzed for SFPQ and NONO transcripts by qRT-PCR. The results (mean±SD of 3 biological replicates) are expressed as relative mRNA levels compared to that obtained for CshRNA expressing cells (assigned a value of 1). **B.** MDA-MB-436/tet-MUC1shRNA cells treated with vehicle or DOX for 7 days were analyzed for the indicated transcripts by qRT-PCR. The results (mean±SD of 6 determinations) are expressed as relative levels compared to that obtained for vehicle-treated cells (assigned a value of 1). **C.** BT-549/CshRNA and BT-549/NF-κBshRNA cells were analyzed for SFPQ and NONO transcripts by qRT-PCR. The results (mean±SD of 3 replicates) are expressed as relative mRNA levels compared to that obtained for the CshRNA expressing cells (assigned a value of 1). **D.** BT-549/CshRNA and BT-549/MUC1shRNA#2 cells were analyzed for the indicated transcripts by qRT-PCR. The results (mean±SD of 3 biological replicates) are expressed as relative mRNA levels compared to that obtained for CshRNA expressing cells (assigned a value of 1). **E.** MDA-

MB-436/tet-MUC1shRNA cells treated with vehicle or DOX for 7 days were analyzed for the indicated transcripts by qRT-PCR. The results (mean±SD of 6 determinations) are expressed as relative levels compared to that obtained for vehicle-treated cells (assigned a value of 1). **F.** BT-549/tet-MYCshRNA cells treated with vehicle or DOX for 7 days were analyzed for RBM14 and HNRNPK transcripts by qRT-PCR. The results (mean±SD of 3 biological replicates) are expressed as relative mRNA levels compared to that obtained for vehicle-treated cells (assigned a value of 1).

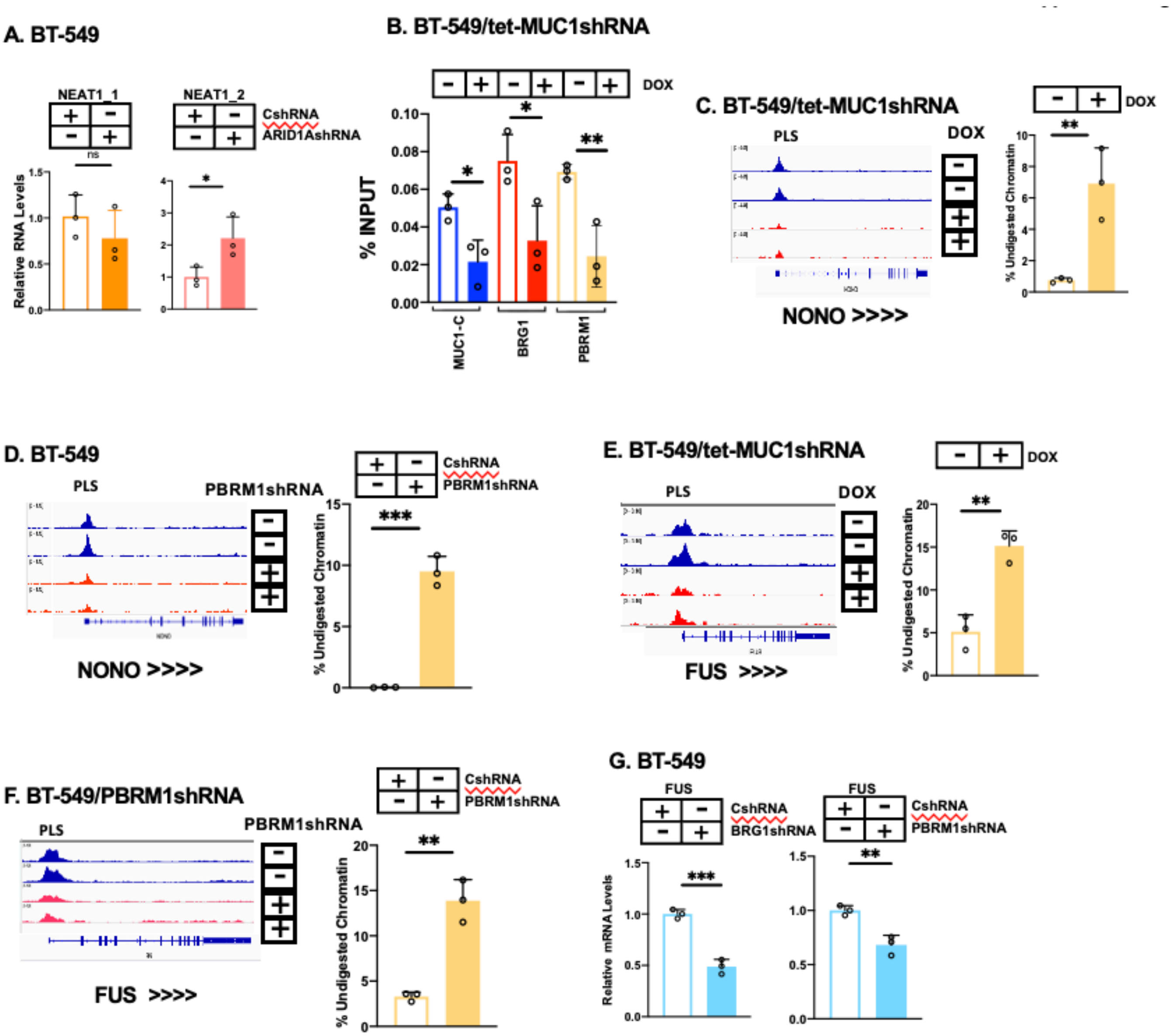


**Supplemental Figure S4. Effects of MUC1-C and PBAF on chromatin accessibility of the *NONO* and *FUS* genes. A.** BT-549 cells expressing a CshRNA or ARID1AshRNA were analyzed for NEAT1_1 and NEAT1_2 transcripts by qRT-PCR. The results (mean±SD of 3 determinations) are expressed as relative RNA levels compared to that obtained for CshRNA expressing cells (assigned a value of 1). **B.** Soluble chromatin from BT-549/tet-MUC1shRNA cells treated with vehicle of DOX for 7 days was precipitated with anti-MUC1-C, anti-BRG1 or anti-PBRM1. The DNA samples were amplified by qPCR with primers for the *NONO* PLS region. The results (mean±SD of 3 replicates) are expressed as percent input. **C.** Genome browser snapshot of ATAC-seq data from the *NONO* PLS in BT-549/tet-MUC1shRNA cells treated with vehicle or DOX for 7 days (left). Chromatin was analyzed for accessibility by nuclease digestion (right). The results (mean±SD of 3 determinations) are expressed as % undigested chromatin. **D.** Genome browser snapshot of ATAC-seq data from the *NONO* PLS in BT-549/CshRNA and BT-549/PBRM1shRNA cells (left).

Chromatin was analyzed for accessibility by nuclease digestion (right). The results (mean±SD of 3 determinations) are expressed as % undigested chromatin. **E**. Genome browser snapshot of ATAC-seq data from the *FUS* PLS in BT-549/tet-MUC1shRNA cells treated with vehicle or DOX for 7 days. Chromatin was analyzed for accessibility by nuclease digestion (right). The results (mean±SD of 3 determinations) are expressed as % undigested chromatin. **F**. Genome browser snapshot of ATAC-seq data from the *FUS* PLS in BT-549/CshRNA and BT-549/PBRM1shRNA cells. Chromatin was analyzed for accessibility by nuclease digestion (right). The results (mean±SD of 3 determinations) are expressed as % undigested chromatin. **G**. BT-549 cells expressing a CshRNA, BRG1shRNA or PBRM1shRNA were analyzed for FUS transcripts by qRT-PCR. The results (mean±SD of 3 biologic replicates) are expressed as relative levels compared to that obtained for CshRNA expressing cells (assigned a value of 1).

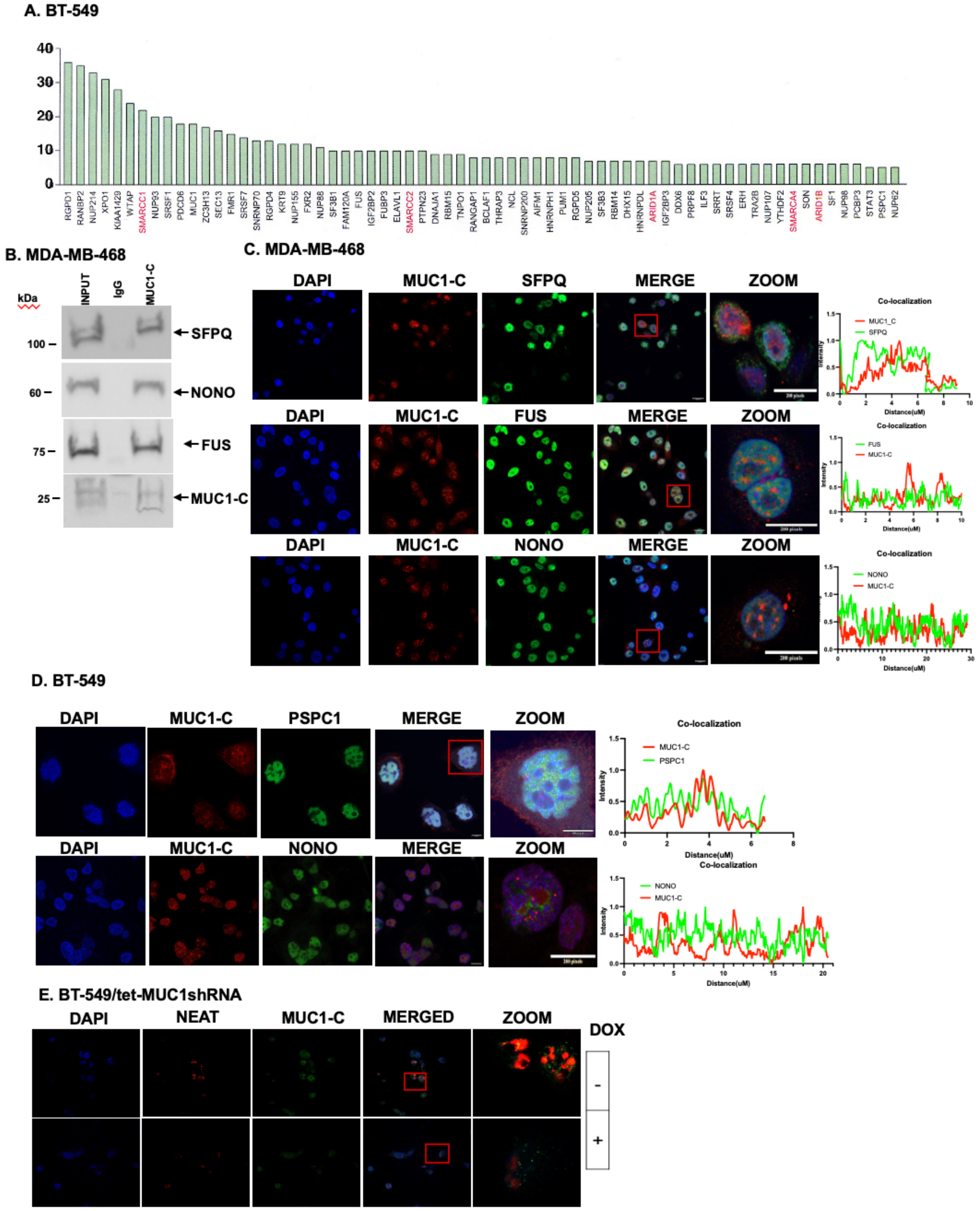


**Supplemental Figure S5. Detection of MUC1-C-associated nuclear proteins and effects of silencing NEAT1 expression. A.** Anti-MUC1-C immunoprecipitates of BT-549 cell nuclear lysates were analyzed by

mass spectroscopy. Shown are the relative intensities of the associated proteins. **B**. Lysates of MDA-MB-468 cells were immunoprecipitated with a control IgG or anti-MUC1-C. The input lysate and precipitates were immunoblotted with antibodies against the indicated proteins. **C**. Immunofluorescence staining of MUC1-C, SFPQ, NONO and FUS in MDA-MB-468 cells. Nuclei were stained with DAPI. The enlarged inset images and colocalization analyses are on the right. Pearson's coefficients of colocalization: MUC1-C+SFPQ=0.779, MUC1-C+FUS=0.769, MUC1-C+NONO=0.797. **D**. Immunofluorescence staining of MUC1-C and PSPC1 and NONO in BT-549 cells. Nuclei were stained with DAPI. The enlarged inset images and colocalization analyses are on the right. Pearson's coefficients of colocalization: MUC1-C+PSPC1=0.702, MUC1-C+NONO=0.542. **E**. Representative NEAT1 RNA FISH and MUC1-C immunofluorescence images of BT-549/tet-MUC1shRNA cells treated with vehicle or DOX for 7 days. Nuclei were counterstained with DAPI.

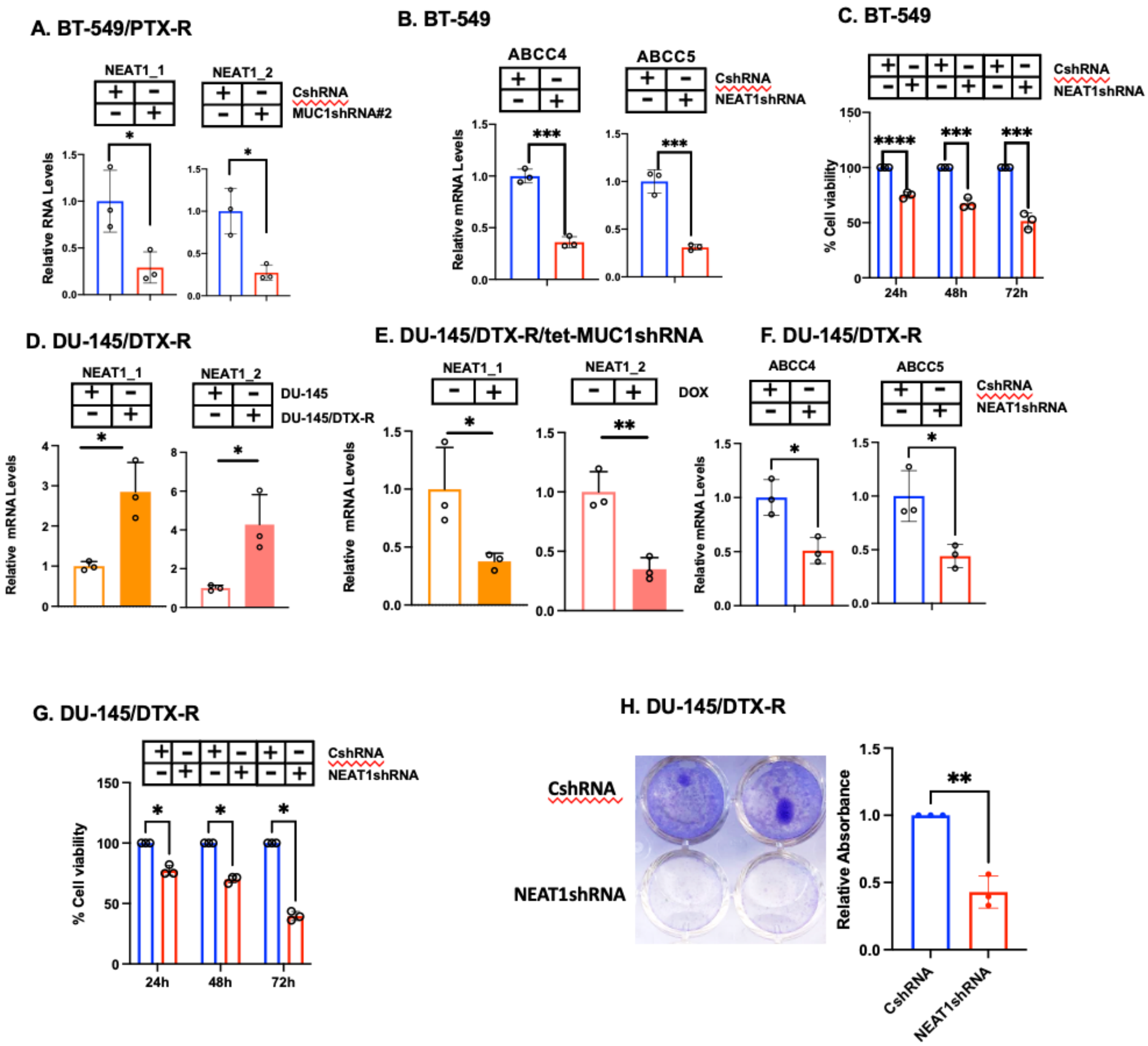


**Supplemental Figure S6. Analysis of NEAT1- and MUC1-C-driven genes in parental BT-549 and BT-549/PTX-R cells. A.** BT-549/PTX-R/CshRNA and BT-549/PTX-R/MUC1shRNA#2 cells were analyzed for NEAT1_1 and NEAT1_2 transcripts by qRT-PCR. The results (mean±SD of three determinations) are expressed as relative levels compared to that obtained for CshRNA cells (assigned a value of 1). **B.** BT-549 cells expressing CshRNA or NEAT1shRNA were analyzed for ABCC4 and ABCC5 transcripts by qRT-PCR. The results (mean±SD of three independent replicates) are expressed as relative levels compared to that obtained for CshRNA cells (assigned a value of 1). **C.** BT-549 cells expressing CshRNA or NEAT1shRNA were treated with 1 nM PTX for 24, 48 and 72 hours. Cell viability was assessed by Alamar blue assay. The results (mean±SD of 3 biologic replicates each with 5 determinations) are expressed as relative levels compared to that obtained for CshRNA cells (assigned a value of 1). **D.** DU-145 and DU-145/DTX-R cells were

analyzed for NEAT1_1 and NEAT1_2 transcripts by qRT-PCR. The results (mean±SD of three replicates) are expressed as relative levels compared to that obtained for DU-145 cells (assigned a value of 1). **E**. DU-145/DTX-R cells expressing tet-MUC1shRNA were treated with vehicle or DOX for 7 days and analyzed for NEAT1_1 and NEAT1_2 transcripts by qRT-PCR. The results (mean±SD of three replicates) are expressed as relative levels compared to that obtained for vehicle-treated cells (assigned a value of 1). **F**. DU-145/DTX-R cells expressing CshRNA or NEAT1shRNA were analyzed for ABCC4 and ABCC5 transcripts by qRT-PCR. The results (mean±SD of three independent replicates) are expressed as relative levels compared to that obtained for CshRNA cells (assigned a value of 1). **G**. DU-145 cells expressing CshRNA or NEAT1shRNA were treated with 1 nM DTX for 24, 48 and 72 hours. Cell viability was assessed by Alamar blue assay. The results (mean±SD of 3 biologic replicates each with 5 determinations) are expressed as relative levels compared to that obtained for CshRNA cells (assigned a value of 1). **H**. DU-145/DTX-R cells expressing CshRNA or NEAT1shRNA treated with PTX were analyzed for colony formation. Shown are representative photomicrographs of stained colonies (left). The results (mean±SD of three biologic replicates) are expressed as relative absorbance compared to that for untreated cells (assigned a value of 1) (right).

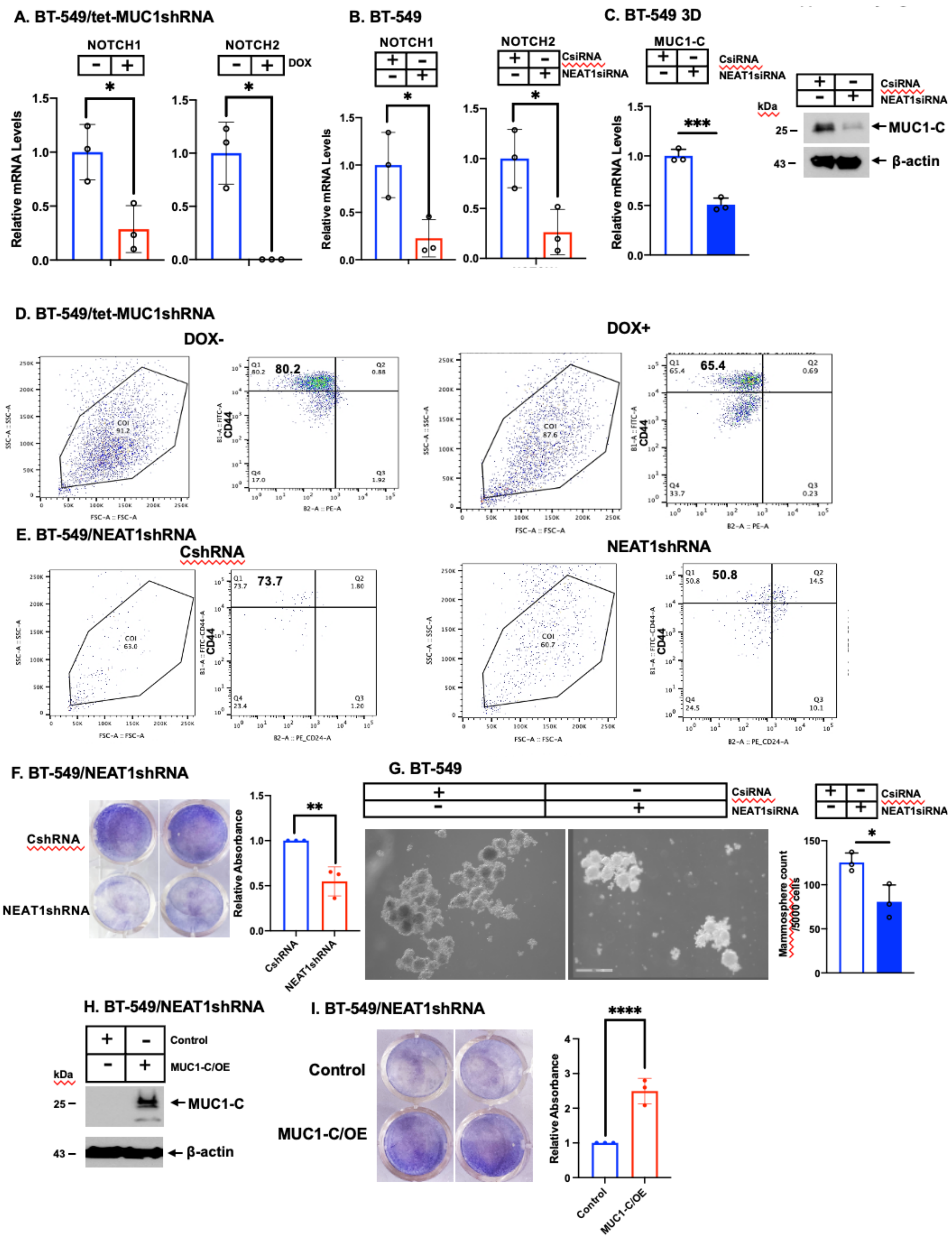
A. BT-549/tet-MUC1shRNA
NOTCH1
NOTCH2
DOX
Relative mRNA Levels
B. BT-549
NOTCH1
NOTCH2
CsiRNA
NEAT1siRNA
Relative mRNA Levels
C. BT-549 3D
MUC1-C
CsiRNA
NEAT1siRNA
Relative mRNA Levels
kDa
25 –
43 –
MUC1-C
β-actin
D. BT-549/tet-MUC1shRNA
DOX-
DOX+
80.2
65.4
CD44
E. BT-549/NEAT1shRNA
CshRNA
NEAT1shRNA
73.7
50.8
CD44
F. BT-549/NEAT1shRNA
CshRNA
NEAT1shRNA
Relative Absorbance
G. BT-549
CsiRNA
NEAT1siRNA
Mammosphere count /5000 cells
H. BT-549/NEAT1shRNA
Control
MUC1-C/OE
kDa
25 –
43 –
MUC1-C
β-actin
I. BT-549/NEAT1shRNA
Control
MUC1-C/OE
Relative Absorbance

**Supplemental Figure S7. Effects of the MUC1-C/NEAT1 pathway on self-renewal capacity. A.** BT-549/tet-MUC1shRNA cells treated with vehicle or DOX for 7 days and were analyzed for the indicated mRNA levels by qRT-PCR. The results (mean±SD of 3 determinations) are expressed as relative levels compared to that obtained for control cells (assigned a value of 1). **B.** BT-549/CsiRNA and BT-549/NEAT1siRNA cells were analyzed for the indicated mRNA levels by qRT-PCR. The results (mean±SD of 3 determinations) are expressed as relative levels compared to that obtained for control cells (assigned a value of 1). **C.** BT-549/CsiRNA 3D and BT-549/NEAT1siRNA 3D cells were analyzed for MUC1-C transcripts by qRT-PCR (left). The results (mean±SD of 3 determinations) are expressed as relative levels compared to that obtained for control cells (assigned a value of 1). Lysates were immunoblotted with antibodies against the indicated proteins (right). **D.** BT-549/tet-MUC1shRNA cells treated with vehicle or DOX for 7 days were analyzed for cell surface expression of CD44 by flow cytometry. Indicated are the % positive cells. **E.** BT-549/CshRNA and BT-549/NEAT1shRNA cells were analyzed for the cell surface expression of CD44 by flow cytometry. Indicated are the % positive cells. **F.** BT-549/CshRNA 3D and BT-549/NEAT1shRNA 3D cells were analyzed for colony formation. Shown are representative photomicrographs of stained colonies (left). The results (mean±SD of three determinations) are expressed as relative absorbance compared to that for untreated cells (assigned a value of 1) (right). **G.** BT-549/CsiRNA 3D and BT-549/NEAT1siRNA 3D cells were analyzed for tumorsphere formation (left). The results (mean±SD of three determinations) are expressed as tumorsphere number (right). **H.** DU-145/CsiRNA 3D and BT-549/NEAT1siRNA 3D cells were analyzed for tumorsphere formation (right). Photomicrographs are shown for the tumorspheres. The results (mean±SD of three determinations) are expressed as tumorsphere number (right). **I.** Lysates from BT-549/NEAT1shRNA 3D cells transfected with a control or MUC1-C-expressing vector were immunoblotted with antibodies against the indicated proteins. **J.** BT-549/NEAT1shRNA 3D cells transfected with a control or MUC1-C-expressing vector were analyzed for colony formation. Shown are representative photomicrographs of stained colonies (left). The results (mean±SD of three determinations) are expressed as relative absorbance compared to that for untreated cells (assigned a value of 1) (right).

**Supplemental Table S1. Primers used for qRT-PCR analyses.**

| | | |
|---|---|---|
| **NOTCH1** | **FWD** | GGGCTAACAAAGATATGCAG |
| | **REV** | ACTGAACCTGACCGTACAGTTGGCAAAGTGGTCCAG |
| **MUC1-C** | **FWD** | TACCGATCGTAGCCCCTATG |
| | **REV** | CTCACCAGCCCAAACAGG |
| **NEAT1_1** | **FWD** | CTTCCTCCCTTTAACTTATCCATTCAC |
| | **REV** | CTCTTCCTCCACCATTACCAACAATAC |
| **NEAT1_2** | **FWD** | CAGTTAGTTTATCAGTTCTCCCATCCA |
| | **REV** | GTTGTTGTCGTCACCTTTCAACTCT |
| **ACTIN** | **FWD** | GATGAGATTGGCATGGCTTT |
| | **REV** | CACCTTCACCGTTCCAGTTT |
| **SFPQ** | **FWD** | ACAAAGGCAAAGGATTCGGA |
| | **REV** | AGATTACGAACAGAAAGGGCAG |
| **NONO** | **FWD** | ACAGATGCAGTGAAGGCTC |
| | **REV** | CTCGTTCCTTGTGAAATTGCTG |
| **FUS** | **FWD** | CACGGACACTTCAGGCTATG |
| | **REV** | GTAAGACGATTGGGAGCTCTG |
| **RBM14** | **FWD** | GCCCAAGGCCTCTTAATACTTG |
| | **REV** | CTTCTCCATGTGAACAAACGC |
| **HNRNPK** | **FWD** | AGCTCCCGCTCGAATCTGAT |
| | **REV** | CCTCAACTCGCAGTCAAAGTC |
| **MYC** | **FWD** | TTCGGGTAGTGGAAAACCAG |
| | **REV** | AGTAGAAATACGGCTGCACC |
| **NOTCH2** | **FWD** | AAGAAACAGAGGATGACAGG |
| | **REV** | ACTGAACCTGACCGTACATGGTCTGAGTCTTGAACACA |
| **ABCC4** | **FWD** | TGCAAGGGTTCTGGGATAAAG |
| | **REV** | GCTGGATTACTTTGGCACTTTC |
| **ABCC5** | **FWD** | AGACCATCCGAGAAGCATTTG |
| | **REV** | GTTGGACAGAAGGACCGATG |

| | | |
|---|---|---|
| **GAPDH** | **FWD** | CCATGGAGAAGGCTGGGG |
| | **REV** | CAAAGTTGTCATGGATGACC |
| **NEAT1** | **FWD** | TGGCTAGCTCAGGGCTTCAG |
| | **REV** | TCTCCTTGCCAAGCTTCCTTC |
| **MUC1-CD** | **FWD** | TGTCAGTGCCGCCGAAAGAA |
| | **REV** | CTACAAGTTGGCAGAAGTGGCT |
| **IL-6** | **FWD** | CCACTCACCTCTTCAGAACG |
| | **REV** | CATCTTTGGAAGGTTCAGGTTG |
| **DUSP2** | **FWD** | CTGCCGTGTACTTCCTGC |
| | **REV** | TGGTTTTGTCCCCTGTTGG |

**Supplemental Table S2. Primers used for ChIP qPCR analyses.**

| | | |
|---|---|---|
| **NEAT1_PLS** | **FWD** | AGTGATGTGGAGTTAAGGCG |
| | **REV** | CCTGGAAAATAAAGCGTTGGTC |
| **NEAT1_dELS** | **FWD** | AAACAGACCGAAACCCCTC |
| | **REV** | CCACTTCCACTGCTCCTT |
| **SFPQ_dELS1** | **FWD** | AAAGTGCTGGGATTATGGGAG |
| | **REV** | TGGACCTCAAAGAAACTGGAG |
| **SFPQ_dELS2** | **FWD** | AAAGATCCAGGCAGATGACC |
| | **REV** | GGATGGTGATACAGGGTAGAAG |
| **NONO_PLS** | **FWD** | CCCCAAAACAGTCTGAGAGTC |
| | **REV** | AGCCATTTAACCCGACCTG |
| **NONO_dELS** | **FWD** | AGGGTTAGTGCAAAGGCTAC |
| | **REV** | GCGCTGCCATTTAAAGGG |
| **FUS** | **FWD** | GTGCCTGGAACATACTAGGTG |
| | **REV** | ATATGCCAATATCCACCCCAC |
